# A molecular networking-guided strategy for identifying anti-osteoporotic sterols from the soft coral *Sclerophytum humesi*

**DOI:** 10.1039/d6ra08640c

**Published:** 2026-11-02

**Authors:** Bo-Rong Peng, Yun-Shiuan Chen, Ting-Mei Wang, Phuong Vu Luu, Quoc-Vu Pham, Guan-Yi Lai, Hsin-Wei Wang, Lo-Yun Chen, Jui-Hsin Su, Kuei-Hung Lai, Huong-Giang Le, Ngoc-Thac Pham, Tuyet Nhung Tran, Mei-Hsien Lee

**Affiliations:** a Department of Biochemistry and Molecular Cell Biology, School of Medicine, College of Medicine, Taipei Medical University Taipei City 110301 Taiwan; b PhD Program in Clinical Drug Development of Herbal Medicine, College of Pharmacy, Taipei Medical University Taipei 110301 Taiwan; c Graduate Institute of Pharmacognosy, College of Pharmacy, Taipei Medical University Taipei 110301 Taiwan; d Faculty of Applied Sciences, Ton Duc Thang University Ho Chi Minh City 758307 Vietnam; e National Museum of Marine Biology and Aquarium Pingtung 944401 Taiwan; f Traditional Herbal Medicine Research Center, Taipei Medical University Hospital Taipei 110301 Taiwan; g Department of Pharmacognosy and Traditional Pharmacy, School of Pharmacy, University of Medicine and Pharmacy at Ho Chi Minh City Ho Chi Minh City 700000 Vietnam; h Center for Reproductive Medicine and Sciences, Taipei Medical University Hospital Taipei 110301 Taiwan

## Abstract

Marine soft corals represent a rich source of structurally diverse secondary metabolites; however, their potential effects on bone metabolism remain poorly explored. In this study, an integrated molecular networking- and bioactivity-guided strategy was employed to identify potential anti-osteoporotic constituents from the soft coral *Sclerophytum humesi.* LC-MS/MS-based molecular networking was integrated with biological activity data to prioritize bioactivity-associated molecular families for targeted isolation. In a glucocorticoid-induced osteoporosis zebrafish model, fraction SH-E3 exhibited a pronounced ability to restore impaired skeletal mineralization and was therefore prioritized for further chemical investigation. Targeted isolation of SH-E3 afforded one previously undescribed compound (1) together with eight known metabolites (3–10), including six sterols: ergosta-5,24(28)-dien-3-ol (5), ergosta-5,22-dien-3-ol (6), cholesterol (7), gorgosterol (8), (3*β*)-33-norgorgost-5-en-3-ol (9), and ergost-5-en-3-ol (10). Parallel investigation of SH-E7 yielded a second previously undescribed compound (2), structurally related to 1. Evaluation of compounds 1–10 in MG-63 cells revealed that sterols 5–10 enhanced alkaline phosphatase (ALP) activity to varying degrees, whereas compounds 1–4 exhibited relatively weak or negligible effects. These findings indicate that the osteogenic activity of SH-E3 is primarily associated with its sterol-enriched chemical profile. Collectively, this study demonstrates the utility of integrating molecular networking with biological activity for the efficient prioritization of bioactive constituents from chemically complex marine extracts and highlights soft-coral-derived sterols as promising candidates for further investigation of their potential roles in glucocorticoid-induced bone loss.

## Abstract 2

Bioactivity-guided fractionation integrated with LC-MS/MS-based molecular networking facilitated the discovery of osteogenic natural products from the soft coral *Sclerophytum humesi*, with anti-osteoporotic effects validated in a zebrafish model.

## Graphical abstract

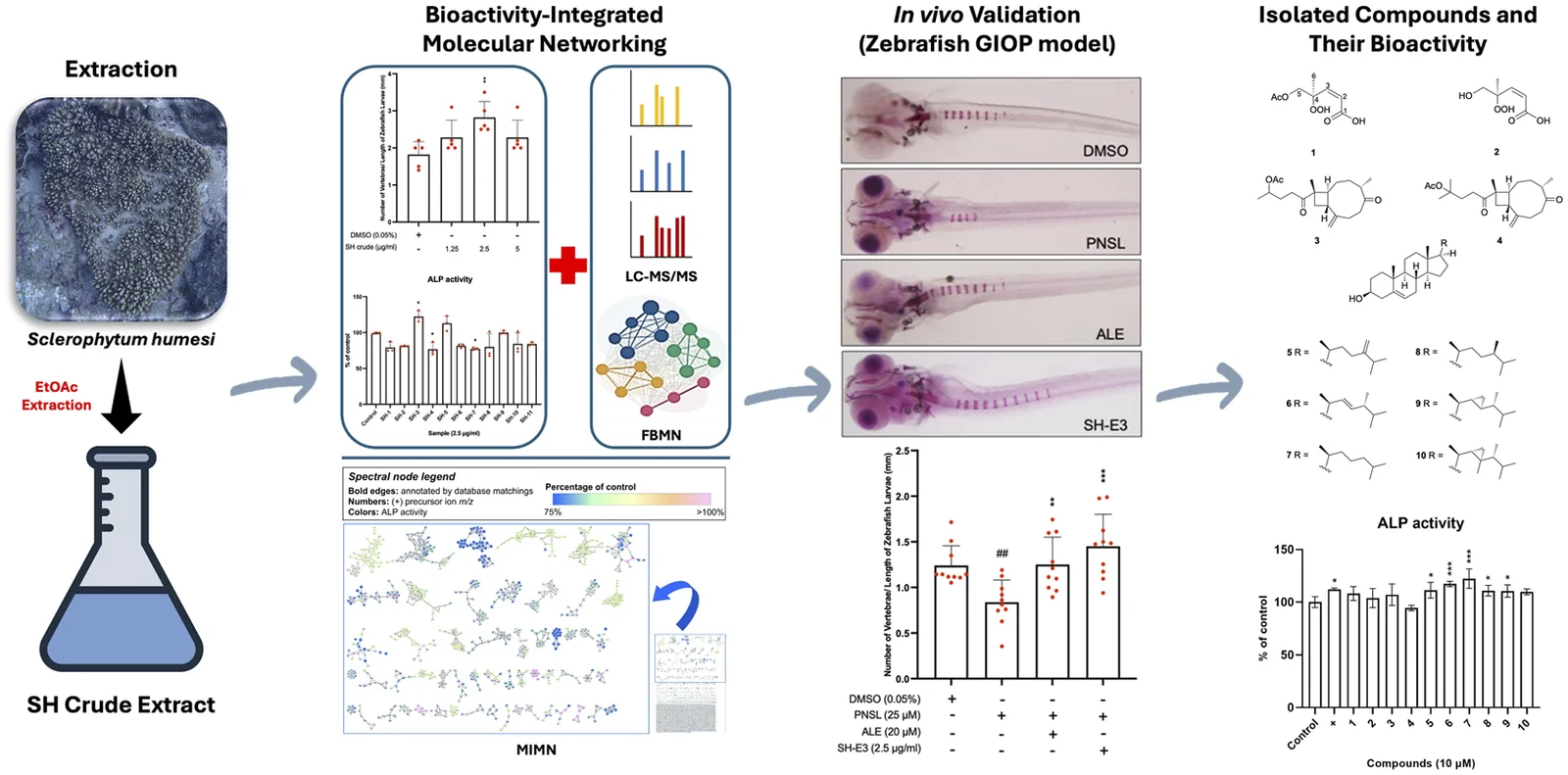

## 1 Introduction

Osteoporosis is a systemic skeletal disorder characterized by reduced bone mass, deterioration of bone microarchitecture, and an increased susceptibility to fragility fractures. Bone homeostasis is maintained through the tightly coordinated activities of bone-forming osteoblasts and bone-resorbing osteoclasts. Disruption of this dynamic balance, particularly through excessive osteoclastogenesis and/or impaired osteoblast differentiation, results in progressive bone loss and ultimately contributes to the development of osteoporosis.^1–3^

Glucocorticoids are widely prescribed for the management of inflammatory and autoimmune diseases; however, prolonged glucocorticoid exposure is a major cause of secondary osteoporosis. Glucocorticoid-induced osteoporosis (GIOP) is characterized by impaired bone remodelling, involving suppression of osteoblast differentiation and bone formation, together with alterations in osteoclast activity and bone-remodelling signalling pathways. Consequently, the identification of bioactive compounds capable of modulating glucocorticoid-induced disturbances in bone homeostasis represents an important strategy for the prevention and management of GIOP.^4–6^

Natural products remain an important source of structurally diverse chemical scaffolds for drug discovery.^7^ Among marine organisms, soft corals are particularly rich in structurally unique secondary metabolites,^8^ including diterpenoids, sesquiterpenoids, steroids, and other terpenoid-derived compounds.^9^ Numerous metabolites isolated from soft corals have demonstrated diverse pharmacological properties, including anti-inflammatory, cytotoxic, and neuroprotective activities.^9–11^ In contrast, the effects of soft-coral-derived metabolites on bone metabolism, particularly their potential protective effects against glucocorticoid-induced bone loss, remain comparatively underexplored.

Despite their considerable chemical diversity, the complexity of marine extracts poses a major challenge to conventional natural-product discovery. Traditional bioactivity-guided fractionation typically requires iterative chromatographic separation and biological evaluation, which is time- and resource-intensive and frequently results in the re-isolation of abundant or previously known metabolites. Mass spectrometry-based molecular networking offers an effective complementary strategy by organizing tandem mass spectrometric features according to their fragmentation similarities, thereby enabling visualization of structurally related molecular families, rapid dereplication, and prioritization of metabolites for targeted isolation. Importantly, integrating molecular networking with biological activity data can further facilitate the identification of bioactivity-associated molecular families directly from complex metabolomic datasets, thereby improving the efficiency of natural-product discovery.^12–14^

*Sclerophytum humesi* is a marine soft coral known to produce structurally diverse secondary metabolites;^15–18^ however, its broader chemical diversity and potential protective effects against glucocorticoid-induced bone loss remain insufficiently investigated. In the present study, we therefore established an integrated natural-product discovery strategy combining LC-MS/MS-based molecular networking, bioactivity-associated molecular prioritization, targeted chromatographic isolation, and an *in vivo* zebrafish model of GIOP. By integrating metabolomic profiles with biological activity data, we aimed to identify bioactive fractions and prioritize molecular families potentially associated with the anti-osteoporotic effects of *S. humesi*. Chemical constituents from the prioritized fraction were subsequently isolated and structurally characterized, and all isolated compounds were evaluated for their effects on alkaline phosphatase (ALP) activity to identify potential anti-osteoporotic constituents.

## 2 Results and discussion

### 2.1 Evaluation of the tolerated concentration of the *S. humesi* extract

Before assessing anti-osteoporotic activity, the potential toxicity of *S. humesi* ethyl acetate (EtOAc) extract (SH) was examined in zebrafish exposed to different concentrations for five days. Survival analysis revealed a concentration-dependent effect of the extract on zebrafish viability. Based on these results, 2.5 µg mL^−1^ was selected as the working concentration for subsequent evaluation of bone mineralization and fraction activity (Fig. 1). This preliminary toxicity assessment was important because apparent alterations in skeletal development at cytotoxic concentrations may result from nonspecific developmental toxicity rather than direct modulation of bone metabolism. The selected concentration therefore provided an appropriate exposure condition for comparing the biological effects of the crude extract and its fractions.

**Fig. 1:**
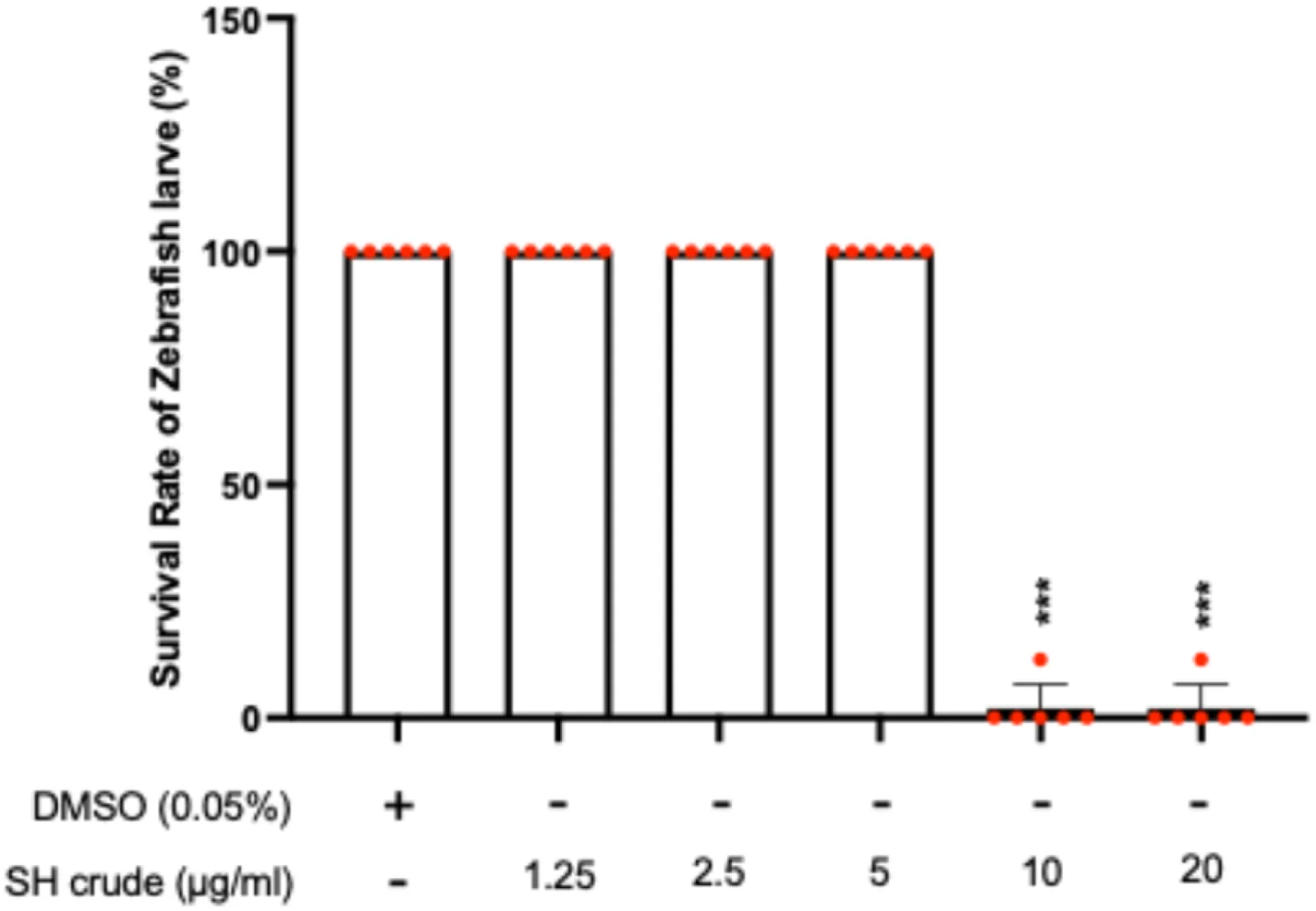
Effect of the crude extract of SH on zebrafish larval viability. Zebrafish larvae were treated with SH crude extract at the indicated concentrations, with 0.05% DMSO used as the vehicle control. Larval viability was assessed after treatment to evaluate the potential toxicity of the crude extract. Data are presented as mean ± SD, with individual data points shown. ***p < 0.001 *versus* the vehicle control.

### 2.2 *S. humesi* extract promotes bone mineralization in zebrafish

The effect of SH on skeletal development was initially evaluated by quantifying vertebral mineralization. Bone mineralization was expressed as the number of calcified vertebrae normalized to body length (N.V./L), thereby minimizing variation caused by differences in overall larval growth. Treatment with SH altered the extent of skeletal mineralization relative to the vehicle-treated group, suggesting that metabolites present in the soft-coral extract may influence bone formation (Fig. 2). These findings provided the biological basis for further fractionation of SH and identification of fractions responsible for its bone-associated activity.

**Fig. 2:**
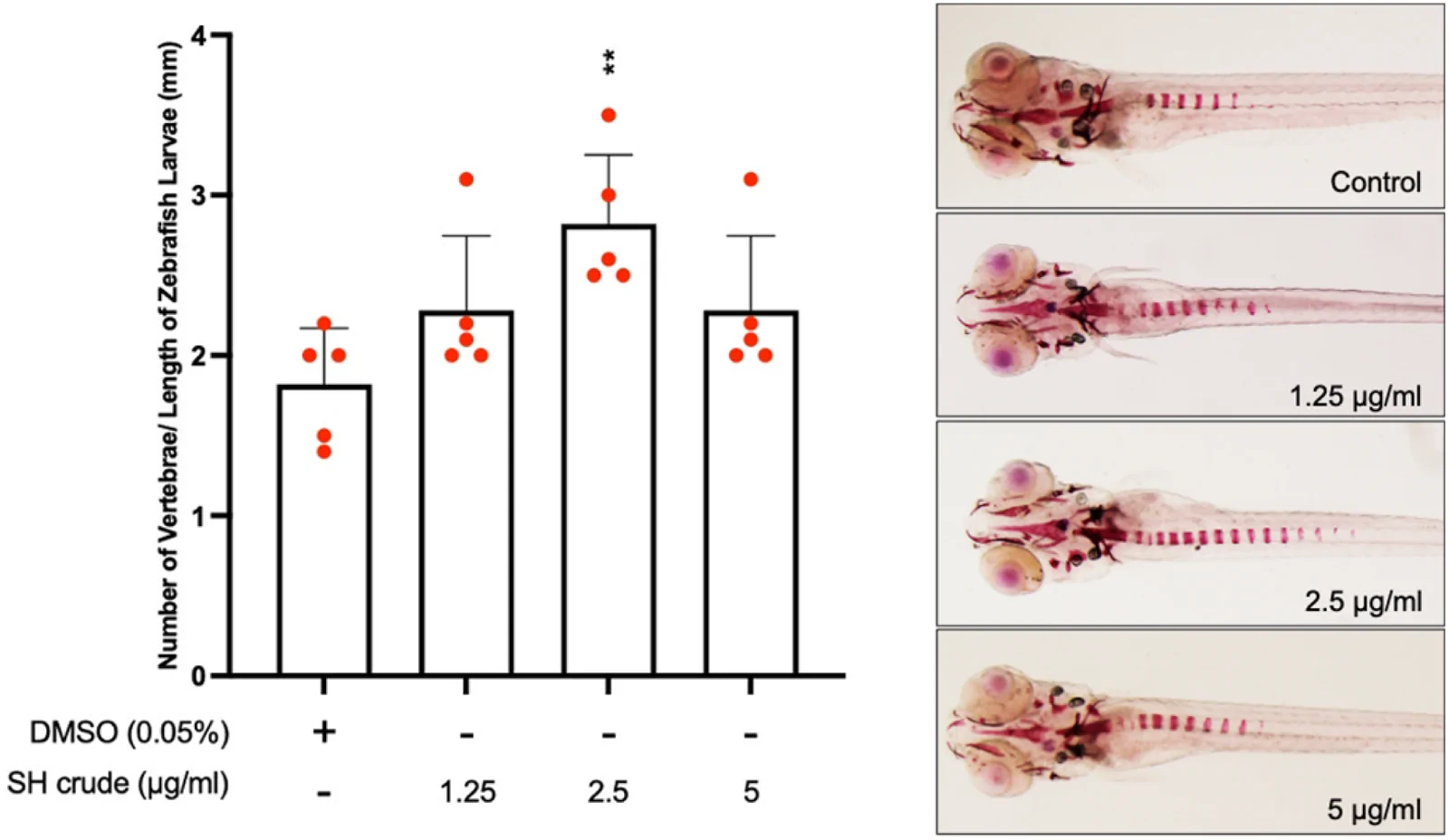
Effects of the SH on skeletal development in zebrafish larvae. Zebrafish larvae were treated with SH at concentrations of 1.25, 2.5, and 5 µg mL^−1^, with 0.05% DMSO used as the vehicle control. Skeletal development was evaluated by measuring the ratio of the number of mineralized vertebrae to larval body length (mm). Representative images of skeletal staining are shown for the control and SH-treated groups. Data are presented as mean ± SD, with individual data points shown (*n* = 5). **p < 0.01 *versus* the vehicle control.

### 2.3 Integration of molecular networking and biological activity prioritizes bioactive *S. humesi* metabolites

To facilitate the prioritization of metabolites potentially responsible for the observed osteogenic activity, LC-MS/MS-based molecular networking was integrated with the *in vitro* ALP activity data obtained from the SH fractions (Fig. S1). Raw LC-MS/MS data were processed using the classical molecular networking workflow on the Global Natural Product Social Molecular Networking (GNPS) platform, in which molecular features exhibiting similar fragmentation patterns were organized into structurally related molecular clusters. Biological activity information was subsequently incorporated into the network to generate a multi-informative molecular network (MIMN), enabling the distribution of ALP-associated features across the SH metabolome to be visualized (Fig. 3A).

**Fig. 3:**
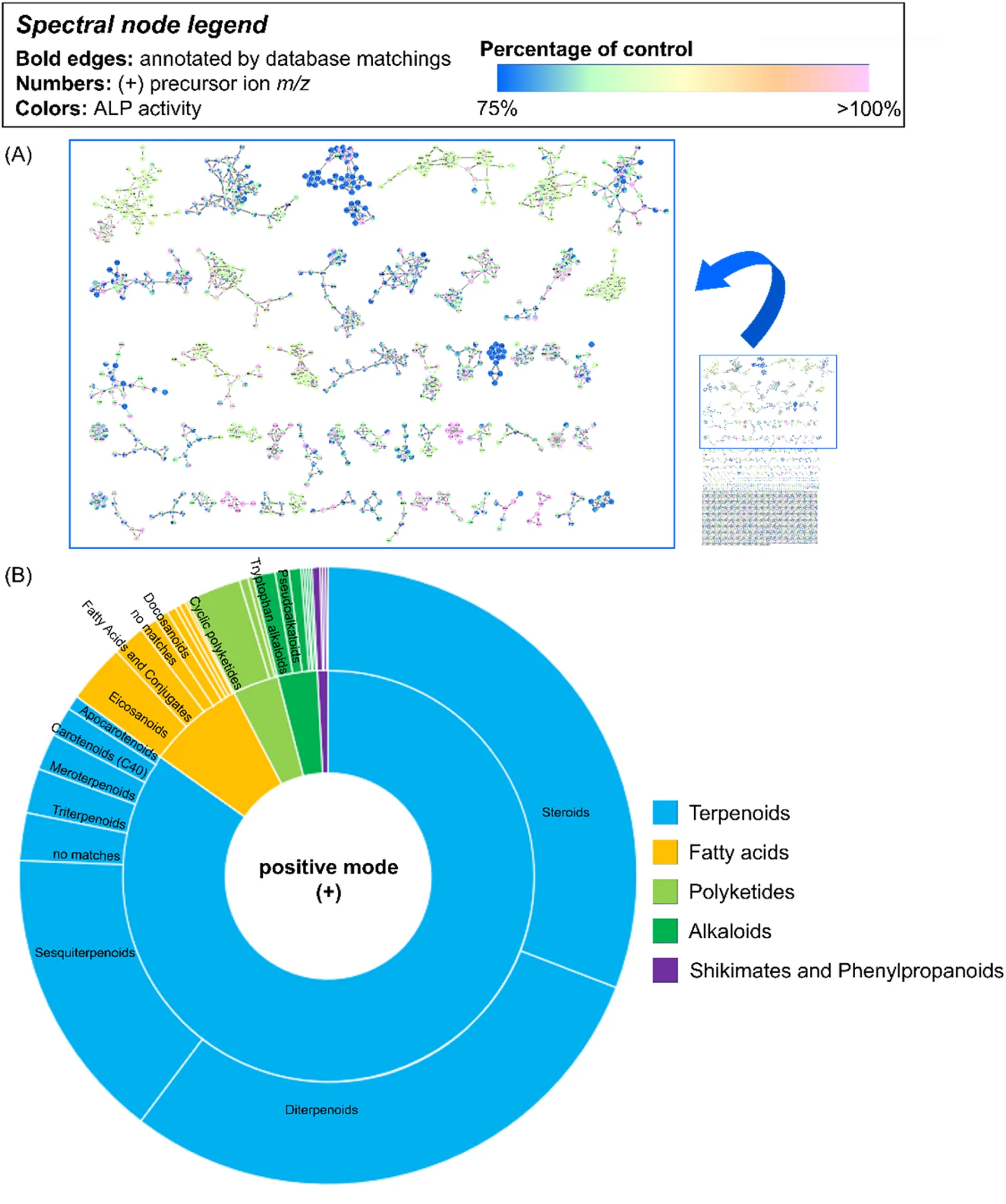
Integration of molecular networking, ALP activity, and chemical classification of SH metabolites. (A) MIMN generated from LC-MS/MS data and integrated with ALP activity data. Nodes are colored according to their associated ALP-promoting activity. (B) MS2Query-based chemical classification of metabolites detected in the prioritized bioactive fraction SH-E3 in positive ionization mode, revealing steroids, diterpenoids, and sesquiterpenoids as the predominant metabolite classes.

SH-E3 was analyzed using the MS2Query workflow on GNPS2 (task ID: caa5edac5fce43c88dd7853ec1c1d5cc), and the resulting chemical classification was visualized as a sunburst plot (Fig. 3B). Terpenoids/Steroids represented the largest chemical group and were therefore prioritized as candidate bioactivity-associated molecular families for further chemical investigation.

### 2.4 Identification of SH-E3 as a bioactive fraction in the zebrafish GIOP model

Based on the *in vitro* ALP activity assay, SH-E3 exhibited the most pronounced stimulatory effect on osteoblastic activity among the tested fractions and was therefore selected for further evaluation in a prednisolone-induced zebrafish GIOP model. Exposure to 25 µM prednisolone markedly impaired skeletal mineralization, consistent with glucocorticoid-induced suppression of bone formation, while alendronate was included as a positive control. Treatment with SH-E3 at 2.5 µg mL^−1^ substantially restored skeletal mineralization under prednisolone exposure (Fig. 4). Collectively, the consistent activity of SH-E3 across the *in vitro* ALP assay and the *in vivo* zebrafish GIOP model provided the rationale for prioritizing this fraction for subsequent molecular-network-based analysis and targeted chemical investigation.

**Fig. 4:**
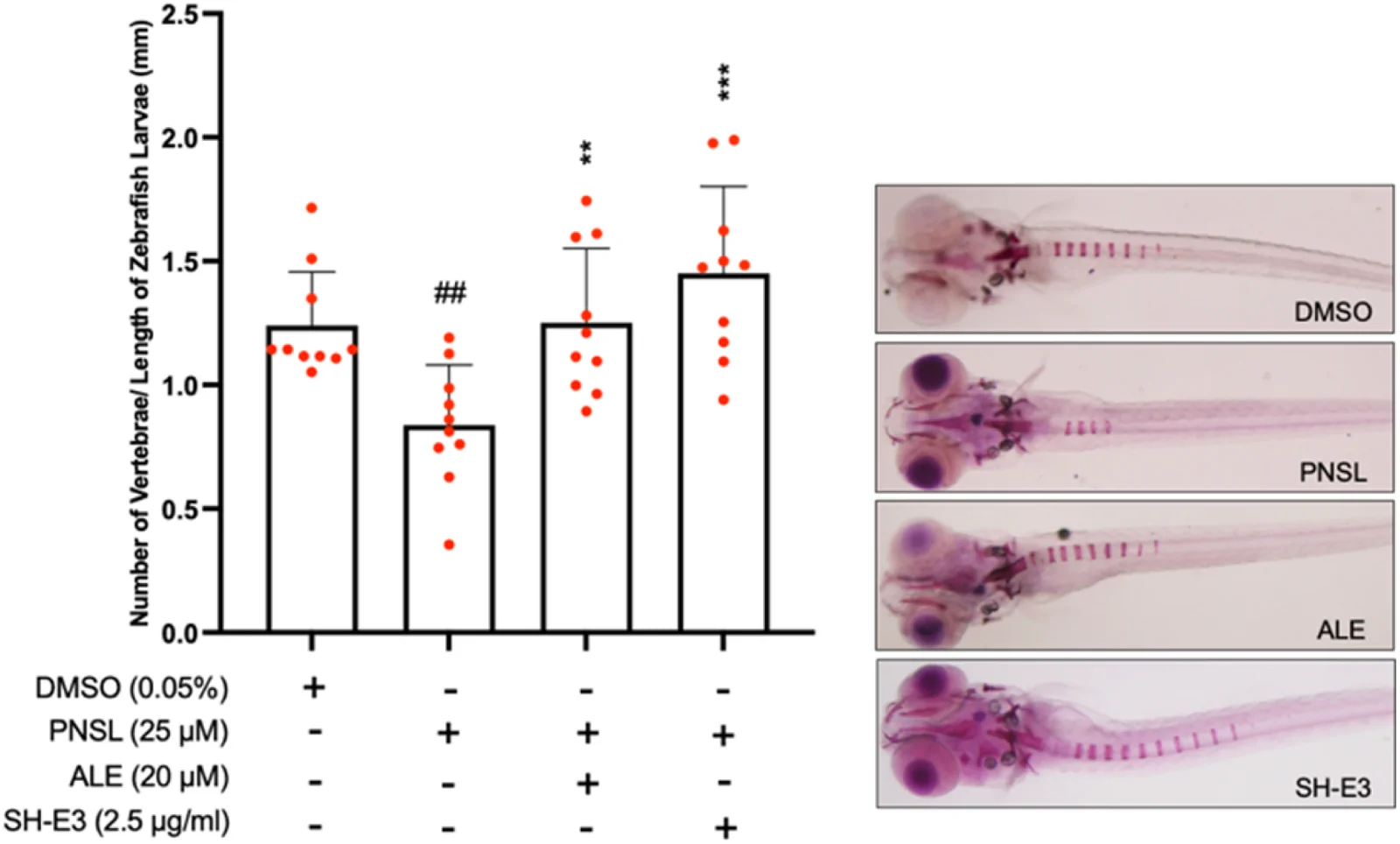
Protective effect of SH-E3 against prednisolone-induced impairment of skeletal mineralization in zebrafish larvae. Zebrafish larvae were exposed to prednisolone (PNSL) to induce GIOP and treated with SH-E3 or alendronate (ALE) as a positive control. Skeletal mineralization was assessed by Alizarin red S staining and quantified as the number of mineralized vertebrae normalized to larval body length. Representative images of Alizarin red S-stained larvae are shown on the right. Data are presented as mean ± SD, with individual data points shown. Statistical significance was determined by one-way ANOVA followed by Dunnett's multiple-comparisons test. **p < 0.01, ***p < 0.001 *versus* the PNSL-treated group; ##*p* < 0.01 *versus* the DMSO control group.

### 2.5 Molecular-network-guided isolation and chemical characterization of *S*. *humesi*

Following biological screening and molecular-network-based prioritization, SH-E3 was selected as the primary fraction for targeted isolation. Chromatographic separation of SH-E3 afforded one previously undescribed compound (1) and eight known metabolites (3–10). Among the known metabolites, sterol-type compounds predominated and were identified as ergosta-5,24(28)-dien-3-ol (5), ergosta-5,22-dien-3-ol (6), cholesterol (7), gorgosterol (8), (3*β*)-33-norgorgost-5-en-3-ol (9), and ergost-5-en-3-ol (10). The remaining two known metabolites were identified as gibberosins O (3) and Q (4). Parallel chemical investigation of SH-E7 led to the isolation of a second previously undescribed compound (2), which was structurally related to compound 1 isolated from SH-E3 (Fig. 5). The enrichment of structurally related sterols (5–10) in the bioactive SH-E3 fraction provides a chemical link between the activity-associated molecular-network features and the metabolites obtained through targeted isolation. Notably, the isolation of two structurally related new compounds (1 and 2) from distinct chromatographic fractions further highlights the chemical diversity of *S. humesi*. Given the considerable structural diversity of marine sterols, particularly in their side-chain architectures and substitution patterns, systematic biological evaluation of compounds 1–10 may provide insights into the structural features associated with their osteogenic effects.

**Fig. 5:**
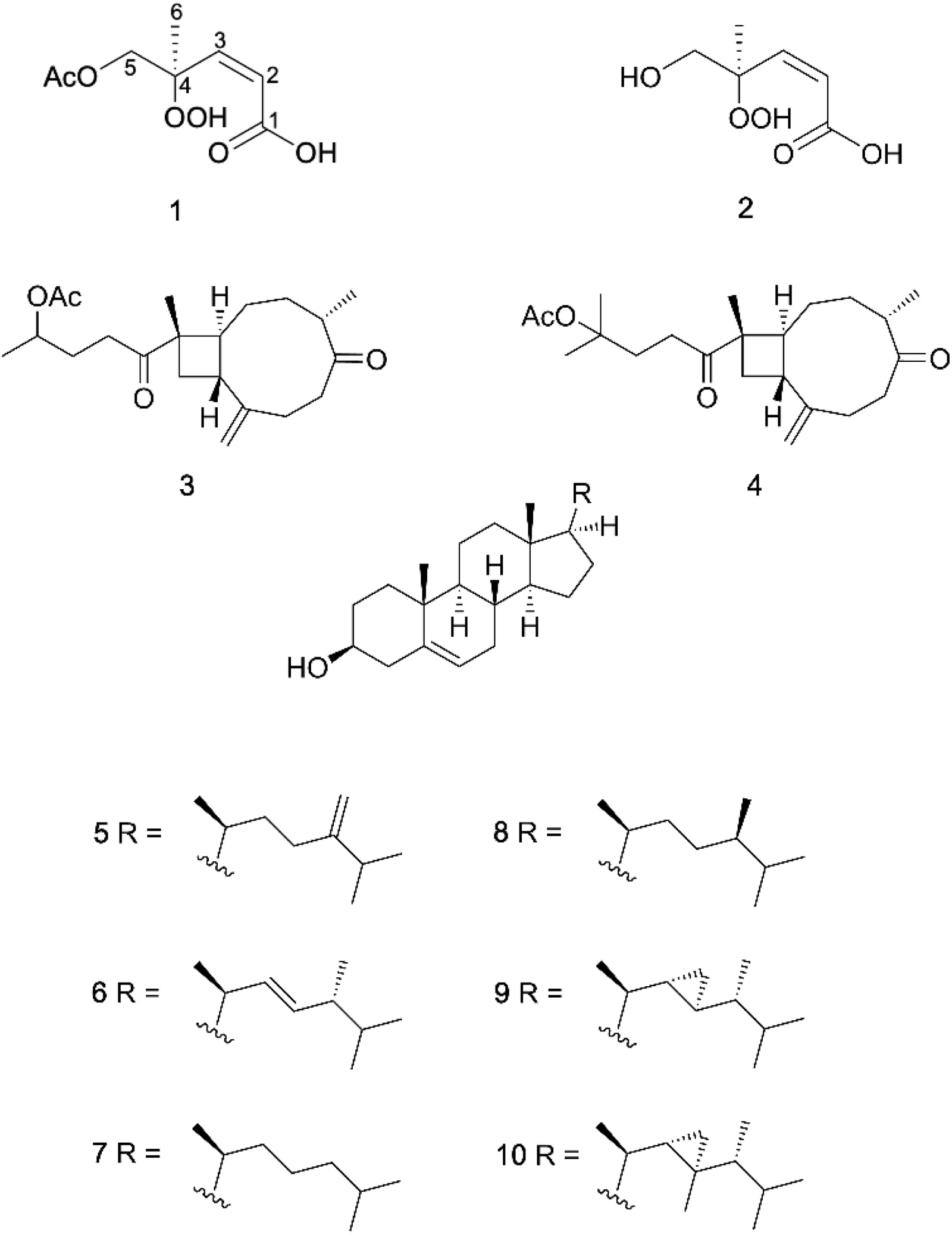
Chemical structures of the isolated compounds (1–10).

### 2.6 Structure elucidation of compounds 1 and 2

Compound 1 was obtained as a colorless oil. Its molecular formula was established as C_8_H_12_O_6_ by HRESIMS, accounting for three degrees of unsaturation. The ^13^C NMR and HSQC spectra revealed eight carbon resonances, including two carbonyl carbons, two olefinic methines, an oxygenated quaternary carbon, an oxygenated methylene, and two methyl carbons (Table 1). The ^1^H NMR spectrum exhibited two mutually coupled olefinic protons at *δ*_H_ 6.09 (d, *J* = 6.0 Hz, H-2) and 7.33 (d, *J* = 6.0 Hz, H-3), an oxygenated methylene at *δ*_H_ 4.32 and 4.16 (each d, *J* = 12.0 Hz, H_2_-5), a methyl singlet at *δ*_H_ 1.49 (H_3_-6), and an acetyl methyl signal at *δ*_H_ 2.03 (s). The ^1^H–^1^H COSY correlation between H-2 and H-3 established the C-2=C-3 unit. Key HMBC correlations from H-2 to C-1 and C-4, from H-3 to C-1 and C-4, and from H_3_-6 to C-3, C-4, and C-5 established the C-1–C-2=C-3–C-4–C-5 carbon framework and located the methyl group at C-4 (Fig. 6). The oxygenated methylene signals at *δ*_H_ 4.32 and 4.16/*δ*_C_ 65.8, together with the HMBC correlation from H_2_-5 to the acetate carbonyl carbon at *δ*_C_ 170.5, established an acetoxy group at C-5. The markedly deshielded quaternary carbon resonance of C-4 (*δ*_C_ 86.9), together with the molecular formula and the remaining oxygen count, supported the presence of a hydroperoxy group at C-4. The *Z* geometry of the C-2/C-3 double bond was assigned from the relatively small coupling constant between H-2 and H-3 (*J* = 6.0 Hz), together with their NOESY correlation (Fig. 6). The absolute configuration at C-4 was further investigated by specific optical rotation (SOR) calculations. The calculated SOR values for the 4*S* and 4*R* configurations were +65 and −68, respectively, whereas compound 1 exhibited an experimental value of −11. The negative sign of the experimental SOR was consistent with that calculated for the 4*R* stereoisomer and opposite to that predicted for the 4*S* stereoisomer, supporting assignment of the 4*R* absolute configuration. Accordingly, the structure of 1 was established as (2*Z*,4*R*)-5-acetoxy-4-hydroperoxy-4-methylpent-2-enoic acid.

**Table 1:** ^1^H and ^13^C NMR spectroscopic data for structures 1 and 2

No.	1		2	
	*δ*_H_ (*J* in Hz)^a^	*δ*_C_ Mult.^b^	*δ*_H_ (*J* in Hz)^a^	*δ*_C_ Mult.^b^
1	—	171.9 C	—	172.6 C
2	6.09 d (6.0)	122.3 CH	6.11 d (5.6)	122.2 CH
3	7.33 d (6.0)	157.0 CH	7.38 d (5.6)	158.2 CH
4	—	86.9 C	—	89.3 C
5	4.32 d (12.0)	65.8 CH_2_	3.69 d (12.0)	66.6 CH_2_
	4.16 d (12.0)		3.80 d (12.0)	
6	1.49 s	20.8 CH_3_	1.47 s	20.2 CH_3_
5-OAc	—	170.5 C	—	—
	2.03 s	20.7 CH_3_	—	—

a Spectrum recorded at 600 MHz in CDCl_3_.

b Spectrum recorded at 150 MHz in CDCl_3_.

**Fig. 6:**
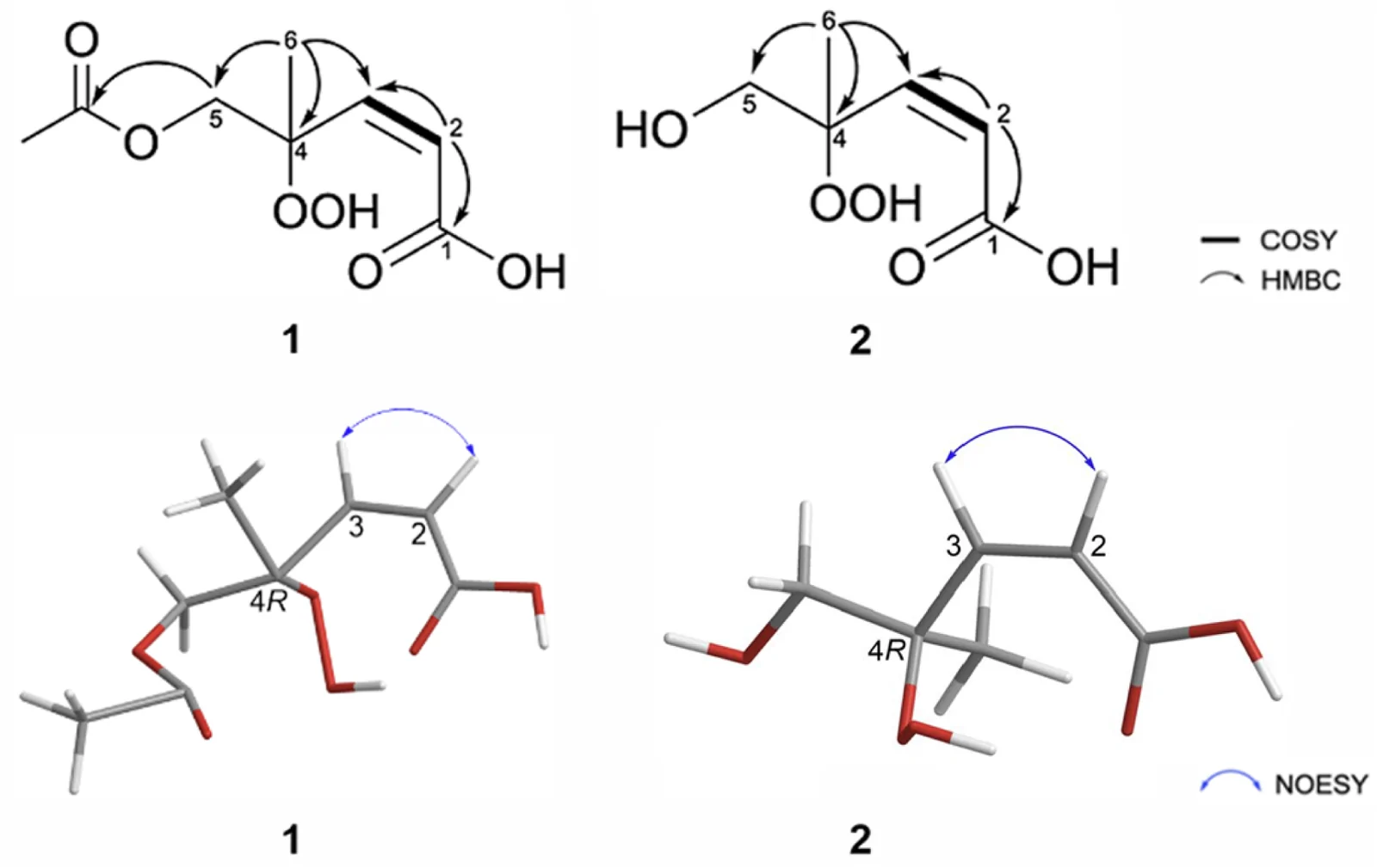
Key COSY, HMBC, and NOESY correlations of compounds 1 and 2.

Compound 2 exhibited NMR spectroscopic features closely resembling those of 1, suggesting that the two compounds possessed the same carbon skeleton. Comparison of their NMR data (Table 1) revealed the absence in 2 of the acetyl carbonyl (*δ*_C_ 170.5) and acetyl methyl (*δ*_H_ 2.03, *δ*_C_ 20.7) resonances observed for 1. In addition, the resonances of the oxygenated methylene at C-5 were shifted from *δ*_H_ 4.32 and 4.16/*δ*_C_ 65.8 in 1 to *δ*_H_ 3.69 and 3.80/*δ*_C_ 66.6 in 2, indicating replacement of the acetoxy group in 1 by a hydroxy group in 2. The ^1^H–^1^H COSY correlation between H-2 and H-3 and the key HMBC correlations from H-2 and H-3 to C-1 and C-4, together with correlations from H_3_-6 to C-3, C-4, and C-5, confirmed that 2 possessed the same C-1–C-2=C-3–C-4–C-5 framework as 1. The oxygenated quaternary carbon at C-4 (*δ*_C_ 89.3), together with the oxygen requirement of the proposed molecular composition, was consistent with a hydroperoxy group at C-4 (Fig. 6). The *Z* configuration of the C-2/C-3 double bond was assigned from the small coupling constant between H-2 and H-3 (*J* = 5.6 Hz), supported by their NOESY correlation (Fig. 6). The absolute configuration at C-4 was further investigated by SOR calculations. The calculated SOR values for the 4*S* and 4*R* configurations were −80 and +80, respectively, whereas compound 2 exhibited an experimental value of +21. The positive experimental SOR value agreed with the calculated value for the 4*R* stereoisomer, supporting the assignment of the absolute configuration at C-4 as 4*R*. Accordingly, the structure of 2 was established as (2*Z*,4*R*)-5-hydroxy-4-hydroperoxy-4-methylpent-2-enoic acid.

### 2.7 Evaluation of ALP-promoting activity of isolated compounds

Subsequent evaluation of compounds 1–10 revealed a distinct activity trend among the isolated metabolites. The sterol-type compounds 5–10 enhanced ALP activity to varying degrees, whereas compounds 1–4 showed relatively weak or negligible effects under the same experimental conditions (Table 2). Among the tested sterols, ergosta-5,22-dien-3-ol (6) and cholesterol (7) exhibited the most pronounced effects, increasing ALP activity to 117.26% and 122.35% of the control, respectively. Compounds 5, 8, and 9 also produced significant increases in ALP activity, whereas compound 10 showed a more modest effect. Notably, none of the tested compounds caused an appreciable reduction in MG-63 cell viability at the tested concentration, suggesting that the observed enhancement of ALP activity was not attributable to nonspecific changes in cell viability.

**Table 2:** Effects of all isolated compounds on ALP activity^a^

	Cell viability	ALP activity
Control	100.00 ± 1.33	100.00 ± 5.17
1	106.44 ± 4.46*	108.14 ± 6.62
2	105.98 ± 1.41	103.85 ± 9.08
3	97.45 ± 0.64	107.09 ± 10.24
4	100.67 ± 3.79	94.42 ± 2.64
5	99.00 ± 5.49	111.31 ± 7.49*
6	99.79 ± 1.87	117.26 ± 2.56***
7	95.74 ± 1.62	122.35 ± 9.30***
8	96.57 ± 1.41	110.84 ± 5.06*
9	98.62 ± 0.47	110.58 ± 5.87*
10	95.78 ± 1.11	109.78 ± 2.80
Rutin (100 µM)	93.94 ± 0.51	112.20 ± 1.09*

a Rutin (100 µM) was used as the positive control. Results are shown as mean ± standard deviation (SD) (*n* = 3), with one-way ANOVA used for statistics (*p < 0.05, ***p < 0.001 *vs.* control).

The preferential activity of compounds 5–10 suggests that the sterol scaffold may represent an important structural feature associated with the ALP-promoting activity of *S. humesi* metabolites. Nevertheless, differences in activity were observed even among these structurally related sterols. In particular, the relatively stronger effects of 6 and 7 compared with 5 and 8–10 suggest that variations in sterol side-chain structure and unsaturation may influence their osteogenic activity. Because the present compound set is limited in both number and structural diversity, however, a definitive structure–activity relationship cannot yet be established, and systematic evaluation of additional sterol analogues will be required to clarify the structural determinants responsible for ALP promotion.

Importantly, this activity pattern is consistent with the chemical composition of the bioactive SH-E3 fraction. Molecular-network-based chemical classification indicated that terpenoid/steroid-related metabolites constituted a major component of SH-E3, and subsequent targeted isolation revealed an enrichment of sterol-type metabolites 5–10 in this fraction. The preferential ALP-promoting effects of these sterols therefore provide biological support for the molecular-network-guided prioritization strategy and establish a chemical–activity link between the metabolomic features of SH-E3 and its observed osteogenic effects. In contrast, the two newly identified compounds 1 and 2, despite expanding the known chemical diversity of *S. humesi*, exhibited only weak effects on ALP activity. Taken together, these findings suggest that the osteogenic activity of SH-E3 is more likely associated with its sterol-enriched chemical profile, rather than with the newly identified compounds 1 and 2 individually.

## 3 Materials and methods

### 3.1 General experimental procedures

The purification and isolation of compounds from the extracts were performed using a series of chromatographic techniques. Flash column chromatography (FCC) was initially conducted using a SepaBean™ machine 2 (China), equipped with either silica gel, to facilitate preliminary fractionation. Subsequently, HPLC was employed to purify the target constituents. Semipreparative HPLC was carried out on a Shimadzu LC-2050 system (Shimadzu, Kyoto, Japan), utilizing a Galaksil EF-C18-H reverse-phase column (120 Å, 5 mm, 10 × 250 mm) to achieve high resolution separation and isolation of the bioactive compounds. Structural elucidation and characterization of the isolated compounds were conducted using a comprehensive suite of spectroscopic techniques. NMR spectroscopy was performed on an Agilent 600 MHz DD2 NMR spectrometer (USA), incorporating both one-dimensional (1D) and two-dimensional (2D) experiments, with chloroform-*d* used as the internal lock solvent to ensure spectral reliability. HR-ESI-MS data were obtained utilizing a SHIMADZU LCMS-9030 system (Japan). IR spectra were recorded employing an IRAffinity-1S FTIR Spectrometer (Japan). UV spectra were measured using a U-3310 UV-Vis spectrophotometer (Japan). ECD spectra were obtained with a JASCO J-715 spectropolarimeter, and optical rotation values were determined in chloroform using a JASCO P-2000 polarimeter (Japan). MG-63 cell lines obtained from the Bioresource Collection and Research Center (Taiwan).

### 3.2 Animal material

Specimens of the soft coral *Sclerophytum humesi* (voucher specimen no. SH-2023) were collected by scuba diving at a depth of approximately 10–15 m off the coast of Pingtung County, Taiwan, in November 2023. The collected specimens were immediately frozen to preserve their chemical constituents and sample integrity until further processing. A voucher specimen was deposited in the herbarium of the College of Pharmacy, Taipei Medical University, Taipei, Taiwan.

### 3.3 Extraction and isolation

The freeze-dried specimen of *Sclerophytum humesi* (300 g) was exhaustively extracted with EtOAc at room temperature. The combined extracts were concentrated under reduced pressure to afford a brown residue (SH, 18.1 g). SH was subjected to silica gel FCC using a stepwise gradient of *n*-hexane (Hex)/EtOAc from 100 : 0 to 0 : 100 at a flow rate of 20 mL min^−1^ over 180 min, yielding 11 fractions (SH-E1–SH-E11). All fractions were subsequently screened for their osteogenic activity using an ALP activity assay. Among them, fraction SH-E3 exhibited the most pronounced ALP-enhancing activity and was therefore selected for further purification. Fraction SH-E3 (600.5 mg) was subjected to silica gel MPLC using a gradient of Hex/EtOAc/MeOH (100 : 0 : 0 to 0 : 80 : 20) to afford two subfractions, SH-E3-A (164 mg) and SH-E3-B (58.7 mg). SH-E3-A was further separated by normal-phase HPLC (NP-HPLC) using Hex/EtOAc (80 : 20) to yield E3-A2 (3.6 mg), E3-A3 (6.1 mg), and E3-A4 (54.5 mg). E3-A2 was purified by reversed-phase HPLC (RP-HPLC) using acetonitrile (ACN)/H_2_O (60 : 40) to afford 4 (1.60 mg), whereas E3-A3 was purified by RP-HPLC using ACN/H_2_O (58 : 42) to yield 3 (2.80 mg). Further purification of E3-A4 by RP-HPLC using ACN/H_2_O (85 : 15) afforded 5 (21.1 mg), 6 (2.61 mg), 7 (2.55 mg), 8 (3.06 mg), 9 (2.29 mg), and 10 (1.43 mg). In parallel, SH-E3-B was purified by RP-HPLC using ACN/H_2_O (37 : 63) to afford 1 (9.45 mg).

In addition, fraction SH-E7 (320.5 mg) was subjected to RP-HPLC using ACN/H_2_O (32 : 68) to afford seven subfractions (SH-E7A∼SH-E7G). Subsequent purification of SH-E7-A (22.9 mg) by RP-HPLC using MeOH/H_2_O (3 : 97) afforded 2 (4.65 mg).

#### 3.3.1 (2*Z*,4*R*)-5-Acetoxy-4-hydroperoxy-4-methylpent-2-enoic acid (1)

Colorless oil; [α]^27^_D_ −11 (*c* 0.11, CHCl_3_); IR (ATR) *ν*_max_ 3441, 2360, 2338, 1744 cm^−1^; UV (CHCl_3_): *λ*_max_ (log *ε*) = 225 nm (4.98); ^1^H (600 MHz, CDCl_3_) and ^13^C (150 MHz, CDCl_3_) NMR spectroscopic data, Table 1; HRESIMS *m*/*z* 203.0565 [M − H]^−^ (calcd for C_8_H_11_O_6_, 203.0556).

#### 3.3.2 (2*Z*,4*R*)-5-Hydroxy-4-*hydroperoxy*-4-methylpent-2-enoic acid (2)

Colorless oil; [α]^27^_D_ +21 (*c* 0.12, CHCl_3_); IR (ATR) *ν*_max_ 3364, 1736, 1643 cm^−1^; UV (CHCl_3_): *λ*_max_ (log *ε*) = 233 nm (5.02); ^1^H (600 MHz, CDCl_3_) and ^13^C (150 MHz, CDCl_3_) NMR spectroscopic data, Table 1; HRESIMS *m*/*z* 129.0559 [M − H-O_2_]^−^ (calcd for C_6_H_9_O_3_, 129.0552).

### 3.4 Molecular networking and chemical classification analysis

#### 3.4.1 Molecular networking analysis

MIMN was generated using the molecular networking workflow on the GNPS2 platform (task ID: 2d731d7871904a02a009d04e0db3c679).^19^ The resulting molecular network was imported into Cytoscape 3.8.2 for visualization. Nodes were colored according to the corresponding bioactivity of each fraction to facilitate the identification of molecular families potentially associated with biological activity.

#### 3.4.2 MS2Query-based chemical classification

MS/MS spectra were processed using MZmine 4.7.8 and exported in Mascot Generic Format (MGF). The resulting MGF file was uploaded to the GNPS2 platform and analyzed using the MS2Query workflow for chemical classification.^20^ The MS2Query results were filtered according to the model prediction score and precursor *m*/*z* difference. Features with model prediction scores greater than the median value and precursor *m*/*z* difference values <50 were retained, resulting in 687 features. Chemical classifications generated by NPClassifier were subsequently used to summarize the chemical composition and construct the sunburst plot shown in Fig. 1B.^21^

### 3.5 Zebrafish experiment

#### 3.5.1 Zebrafish maintenance

Wild-type AB strain zebrafish (*Danio rerio*) were maintained in a 90 × 60 × 45 cm aquarium containing dechlorinated water at 28 ± 0.5 °C and pH 7.0 ± 0.5 under a 14 h light/10 h dark photoperiod. Adult fish were obtained from the Taiwan Zebrafish Center. All animal procedures were performed in accordance with the Guidelines for Care and Use of Laboratory Animals of Taipei Medical University and approved by the Animal Ethics Committee of LAC-2020-0449. Fertilized embryos were cultured in E3 medium composed of 5 mM NaCl, 0.33 mM MgSO_4_, 0.17 mM KCl, and 0.3 mM CaCl_2_ (Sigma-Aldrich, St. Louis, MO, USA) prepared with deionized water. Larvae were not fed during the experimental period, and the medium was refreshed daily.^22^

#### 3.5.2 Glucocorticoid treatment

Larvae were distributed into 24-well plates at a density of six larvae per well. From 3 days post-fertilization (dpf) to 8 dpf, larvae were exposed to drug-containing E3 medium. From 5 dpf onward, larvae were co-treated with 25 µM prednisolone, while 0.05% (v/v) DMSO served as the vehicle control. Treatment solutions were renewed daily, and cumulative survival rates were recorded at 8 dpf.^22,23^

#### 3.5.3 Alizarin red S staining

Larvae were euthanized with 0.1 mg mL^−1^ tricaine methane sulfonate (MS-222) and fixed in 4% paraformaldehyde at 28 °C for 2 h. Pigmentation was removed using bleaching buffer (3% H_2_O_2_, 0.5% KOH), followed by washing in wash buffer (10% glycerol, 0.5% KOH). Samples were stained with 0.02% Alizarin red S in staining buffer (10% glycerol, 0.5% KOH) at 28 °C overnight. Excess stain was removed using destaining buffer (50% glycerol, 0.5% KOH), and specimens were imaged under a stereomicroscope.^22,23^

#### 3.5.4 Vertebral development rate

Vertebral calcification was classified into three categories: fully calcified (100%), dorsal and ventral calcification (67%), and unilateral calcification (33%). The vertebral development rate was calculated as the number of calcified vertebrae normalized to larval length (mm), expressed as N.V./L.^22^

### 3.6 ALP activity and cell viability assays

#### 3.6.1 Cell viability assay

Cell viability was evaluated using the MTT assay. MG-63 cells were maintained in α-MEM (Gibco™, Thermo Fisher Scientific, Waltham, MA, USA) supplemented with 10% fetal bovine serum (FBS) at 37 °C in a humidified atmosphere containing 5% CO_2_. Cells were seeded in 96-well plates at a density of 1 × 10^3^ cells per well in 200 µL of culture medium and incubated for 24 h. The medium was then replaced with fresh medium containing the test compounds (10 µM) or rutin (100 µM, positive control), and the cells were incubated for an additional 72 h. Subsequently, 20 µL of MTT solution (5 mg mL^−1^ in PBS) was added to each well, followed by incubation at 37 °C for 4 h. The medium was then carefully removed, and the resulting formazan crystals were dissolved in 100 µL of DMSO. Absorbance was measured at 600 nm using a microplate reader, and cell viability was expressed relative to that of the untreated control group.^24,25^

#### 3.6.2 ALP activity assay

ALP activity was measured to evaluate the effects of the isolated compounds on osteoblastic differentiation. MG-63 cells were seeded in 96-well plates at a density of 1 × 10^3^ cells per well in 200 µL of culture medium and incubated for 24 h. The cells were subsequently treated with the test compounds (10 µM) or rutin (100 µM, positive control) for an additional 72 h. Following treatment, the cells were washed once with physiological saline and lysed with 1% Triton X-100. ALP activity was determined by incubating the cell lysates with 15 mM *p*-nitrophenyl phosphate (*p*NPP) at 37 °C for 1 h. The formation of *p*-nitrophenol was quantified by measuring the absorbance at 405 nm using a microplate reader. Total protein content was determined using the Pierce™ BCA Protein Assay Kit (Thermo Fisher Scientific, Waltham, MA, USA) according to the manufacturer's instructions. ALP activity was normalized to the total protein content.^24,25^

### 3.7 Statistical analysis

Statistical analyses were performed using GraphPad Prism (GraphPad Software, San Diego, CA, USA). Data are presented as the mean ± standard deviation (SD) from three independent experiments (*n* ≥ 3), with each experiment performed in triplicate. Statistical differences among groups were evaluated by one-way analysis of variance (ANOVA), followed by an appropriate post hoc multiple-comparison test against the control group. A *p* value < 0.05 was considered statistically significant.

### 3.8 *In silico* calculations

All calculations were performed using Gaussian 16 (Gaussian, Inc., Wallingford, CT, USA). Preliminary conformational searches were conducted using the GMMX Conformer Dialog, and the lowest-energy conformers were selected for subsequent calculations. Geometry optimization and frequency calculations were performed using density functional theory (DFT) at the B3LYP/6-31G(d,p) level, with solvent effects modeled using the integral equation formalism polarizable continuum model (IEFPCM) for CHCl_3_. SOR calculations were subsequently performed at the B3LYP/6-31G(d) level using the same solvent model.^24^

## 4 Conclusions

In this study, an integrated molecular networking- and bioactivity-guided strategy was established to investigate the anti-osteoporotic potential of the soft coral *Sclerophytum humesi*. Biological evaluation using a prednisolone-induced zebrafish model of glucocorticoid-induced osteoporosis identified SH-E3 as a bioactive fraction capable of restoring impaired skeletal mineralization. Molecular-network-based prioritization followed by targeted chromatographic isolation led to the identification of ten metabolites, including two previously undescribed compounds (1 and 2) and six sterol-type metabolites (5–10). Subsequent biological evaluation demonstrated that sterols 5–10 enhanced ALP activity to varying degrees, whereas compounds 1–4 exhibited relatively weak or negligible effects, indicating that the osteogenic activity of SH-E3 is primarily associated with its sterol-enriched chemical profile. Collectively, these findings demonstrate the utility of integrating molecular networking with biological activity for the efficient prioritization of bioactive constituents from chemically complex marine extracts and highlight soft-coral-derived sterols as promising candidates for further investigation of their potential roles in bone formation and glucocorticoid-induced bone loss.

## Author contributions

Bo-Rong Peng: conceptualization, investigation, analysis of results, methodology, supervision, visualization, writing – original draft, writing-reviewing and editing. Yun-Shiuan Chen: investigation, analysis of results, data curation. Ting-Mei Wang: investigation, methodology. Phuong Vu Luu: investigation, analysis of results. Quoc-Vu Pham: investigation, data curation. Guan-Yi Lai: analysis of results. Hsin-Wei Wang: data curation. Lo-Yun Chen: investigation, analysis of results. Jui-Hsin Su: methodology. Kuei-Hung Lai: software, analysis of results. Huong-Giang Le: investigation, analysis of results. Ngoc-Thac Pham: investigation, analysis of results. Tuyet Nhung Tran: methodology, analysis of results. Mei-Hsien Lee: conceptualization, supervision, visualization, project administration, writing-reviewing and editing.

## Conflicts of interest

The authors have declared no conflict of interest.

## Supplementary Material

RA-OLF-D6RA08640C-s001

## Data Availability

The data supporting this article have been included as part of the supplementary information (SI). Supplementary information: *in vitro* ALP assay of all fractions, HRESIMS, UV, IR, 1D and 2D NMR spectra of compounds 1–2. See DOI: https://doi.org/10.1039/d6ra08640c.
