# Supplementary material for: A molecular networking-guided strategy for identifying anti-osteoporotic sterols from the soft coral Sclerophytum humesi
Source: RSC Adv. 2026 Nov 2;16(49):67799–807. doi: 10.1039/d6ra08640c (PMC13725852; doi:10.1039/d6ra08640c)
Supplement: Supplementary file 1 [file RA-016-D6RA08640C-s001.pdf]

Supplementary Information (SI) for RSC Advances.
This journal is © The Royal Society of Chemistry

Supporting Information

A molecular networking-guided strategy for identifying anti-osteoporotic sterols from the soft coral *Sclerophytum humesi*

Bo-Rong Peng [†a], Yun-Shiuan Chen [†b], Ting-Mei Wang[c], Phuong Vu Luu[b,c], Quoc-Vu Pham[d], Guan-Yi Lai[c], Hsin-Wei Wang[c], Lo-Yun Chen[b], Jui-Hsin Su[e], Kuei-Hung Lai[b,c,e,f], Huong-Giang Le[g], Ngoc-Thac Pham[g], Tuyet Nhung Tran[d], Mei-Hsien Lee[b,c,h,*]

[a] *Department of Biochemistry and Molecular Cell Biology, School of Medicine, College of Medicine, Taipei Medical University, Taipei City 110301, Taiwan*

[b] *PhD Program in Clinical Drug Development of Herbal Medicine, College of Pharmacy, Taipei Medical University, Taipei, 110301, Taiwan*

[c] *Graduate Institute of Pharmacognosy, College of Pharmacy, Taipei Medical University, Taipei 110301, Taiwan*

[d] *Faculty of Applied Sciences, Ton Duc Thang University, Ho Chi Minh City, Vietnam*

[e] *National Museum of Marine Biology and Aquarium, Pingtung 944401, Taiwan*

[f] *Traditional Herbal Medicine Research Center, Taipei Medical University Hospital, Taipei 110301, Taiwan*

[g] *Department of Pharmacognosy and Traditional Pharmacy, School of Pharmacy, University of Medicine and Pharmacy at Ho Chi Minh City, Ho Chi Minh City 700000, Vietnam*

[h] *Center for Reproductive Medicine and Sciences, Taipei Medical University Hospital, Taipei 110301, Taiwan*

† These authors have contributed equally to this work.

Contents

Figure S1. Effects of Sclerophytum humesi fractions on cell viability and alkaline phosphatase (ALP) activity in MG63 cells. 4

Figure S2. ^{1}H NMR spectrum of **1** (600 MHz, $CDCl_3$). 6

Figure S3. ^{13}C NMR spectrum of **1** (150 MHz, $CDCl_3$). 7

Figure S4 DEPT spectra of **1** (150 MHz, $CDCl_3$). 8

Figure S5. HSQC spectrum of **1.** (600 and 150 MHz, $CDCl_3$). 9

Figure S6. HMBC spectrum of **1**. (600 and 150 MHz, $CDCl_3$). 10

Figure S7. COSY spectrum of **1**. (600 MHz, $CDCl_2$). 11

Figure S8. NOESY spectrum of **1**. (600 MHz, $CDCl_3$). 12

Figure S9. HRESIMS spectrum of **1**. 13

Figure S10. Infrared (IR) spectrum **1**. 14

Figure S11. Ultraviolet (UV) spectrum **1**. 15

Figure S12. ^{1}H NMR spectrum of **2**. (600 MHz, $CDCl_3$). 16

Figure S13. ^{12}C NMR spectrum of **2**. (150 MHz, $CDCl_3$). 18

Figure S14. DEPT spectra of **2**. (150 MHz, $CDCl_3$). 19

Figure S15. HSQC spectrum of ... (**2**) (600 and 150 MHz, $CDCl_3$). 20

Figure S16. HMBC spectrum of **2**. (600 and 150 MHz, $CDCl_3$). 21

Figure S17. COSY spectrum of **2**. (600 MHz, $CDCl_3$). 22

Figure S18. NOESY spectrum of **2**.(600 MHz, $CDCl_3$). 23

Figure S19. HRESIMS spectrum of **2**. 24

Figure S20. Infrared (IR) spectrum **2**. 25

Figure S21. Ultraviolet (UV) spectrum **2**. 26

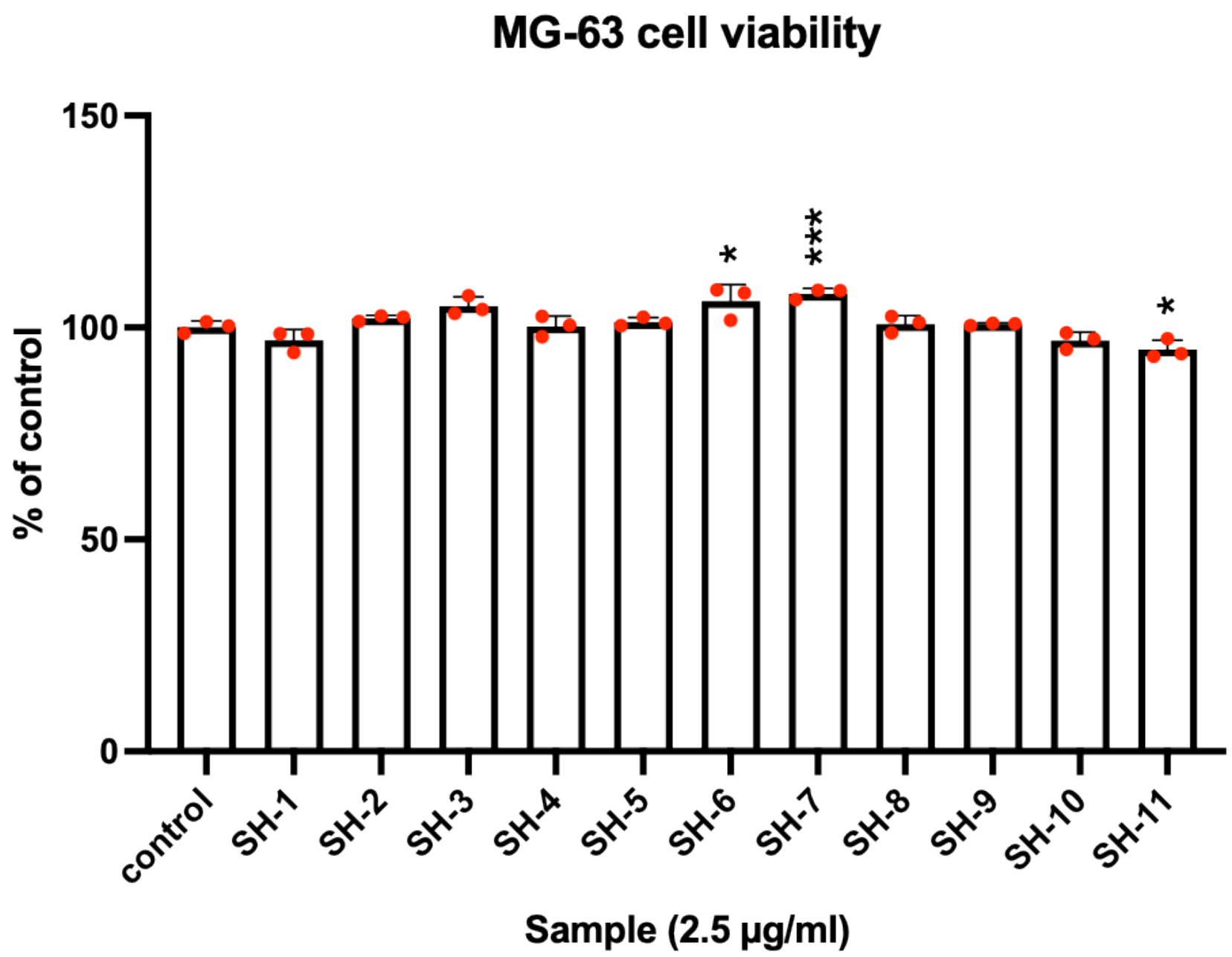

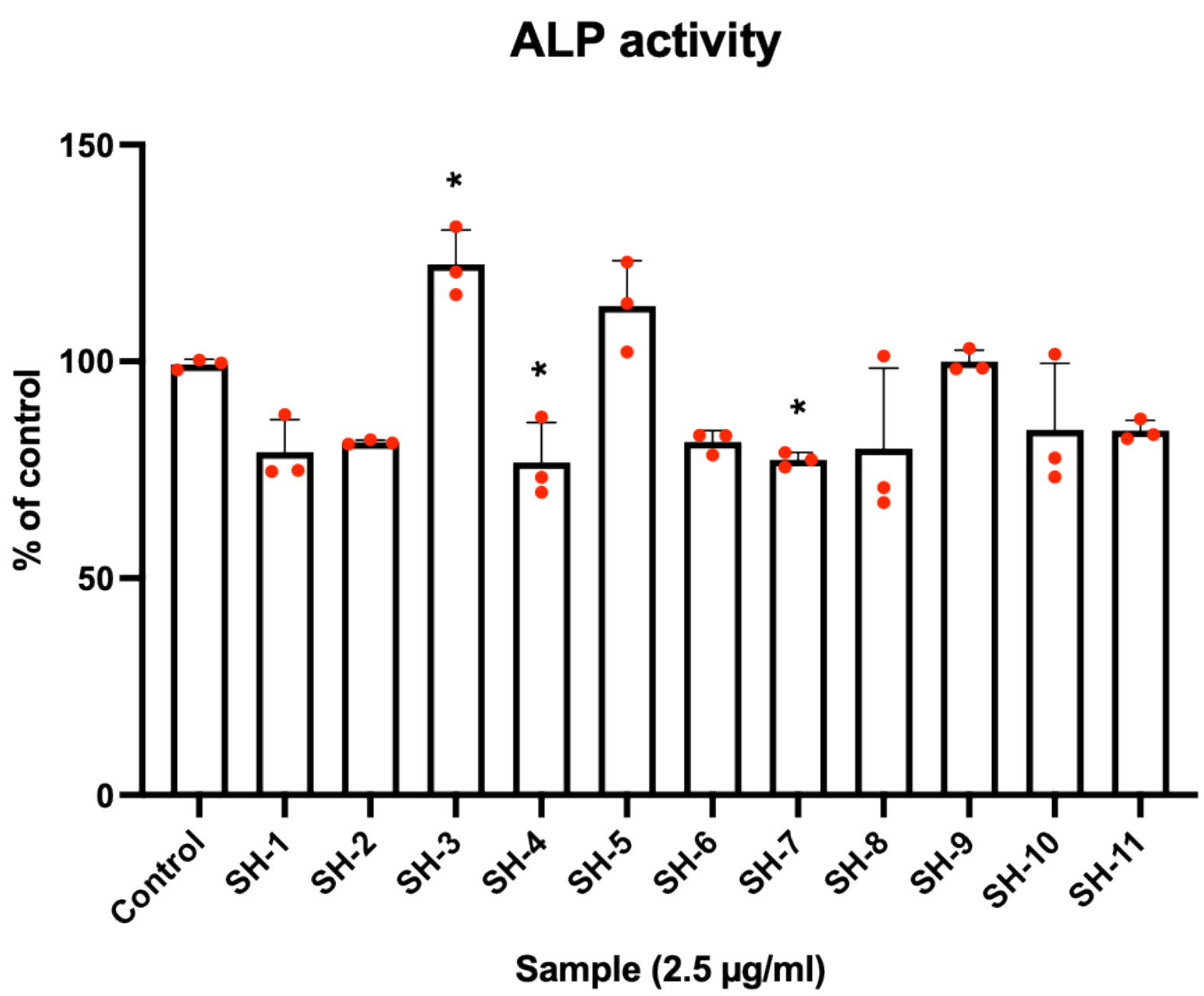

Figure S1. Effects of Sclerophytum humesi fractions on cell viability and alkaline phosphatase (ALP) activity in MG63 cells. (A) Cell viability and **(B)** ALP activity of MG63 cells following treatment with different fractions derived from the EtOAc extract of *S. humesi*. ALP activity was evaluated as an indicator of osteoblastic differentiation. Data are presented as the mean of three independent measurements ($n = 3$), with individual data points shown as red circles.

1. Spectra data for compounds 1 and 2.

1.1. NMR, HRESIMS, IR, and UV spectra of 1.

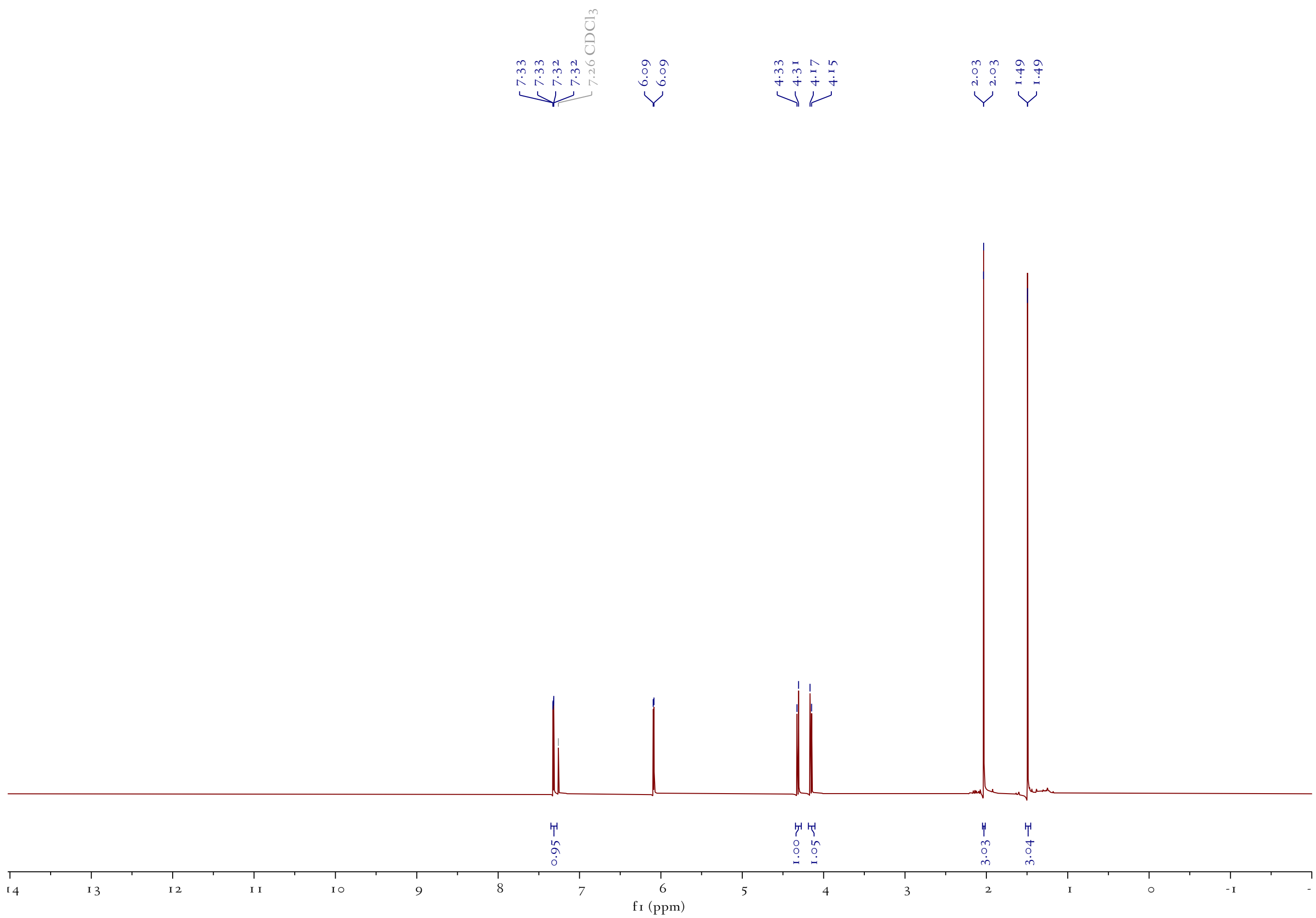

Figure S2. ¹H NMR spectrum of **1** (600 MHz, $CDCl_3$).

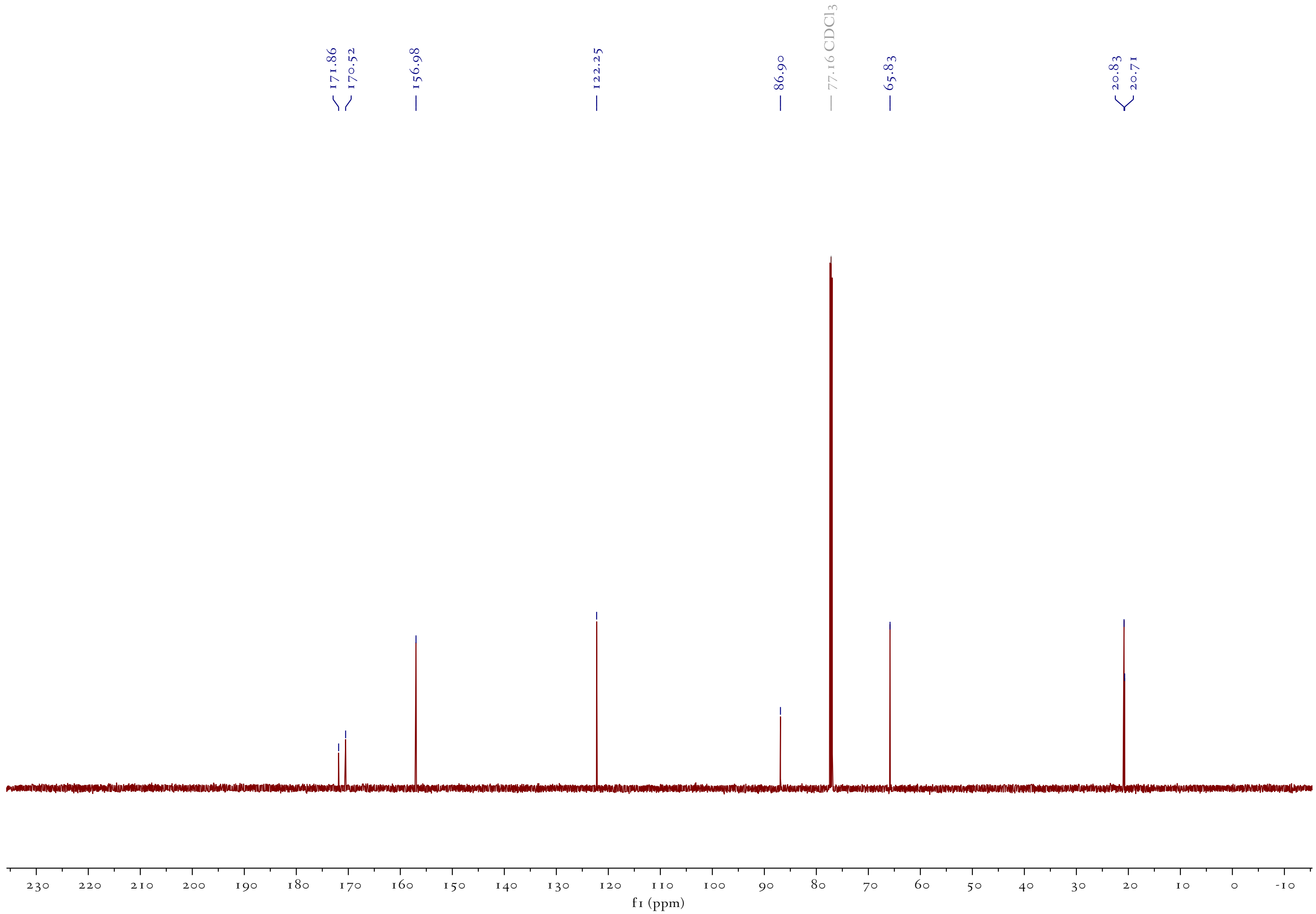

Figure S3. ^{13}C NMR spectrum of **1** (150 MHz, $CDCl_3$).

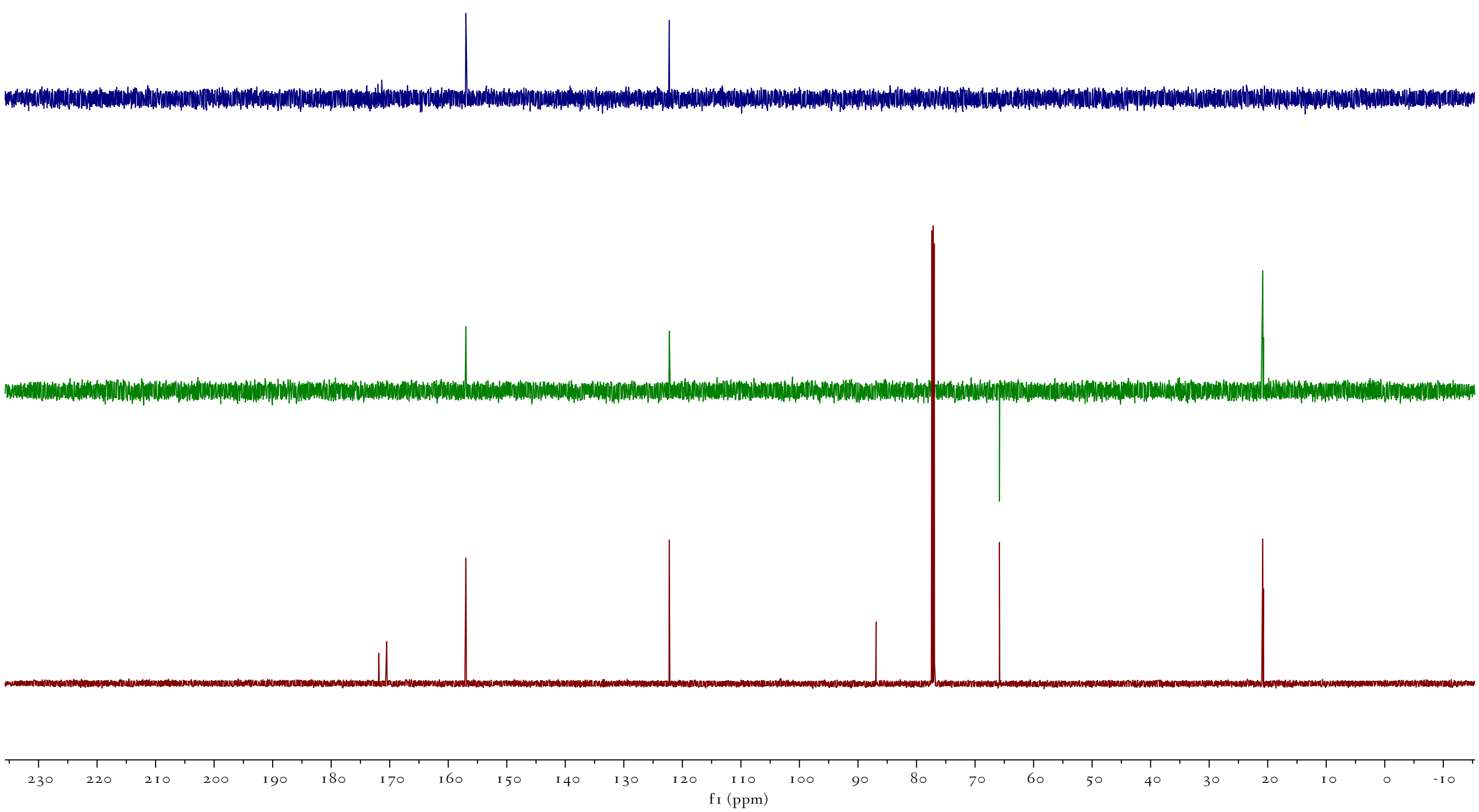

Figure S4 DEPT spectra of **1** (150 MHz, $CDCl_3$).

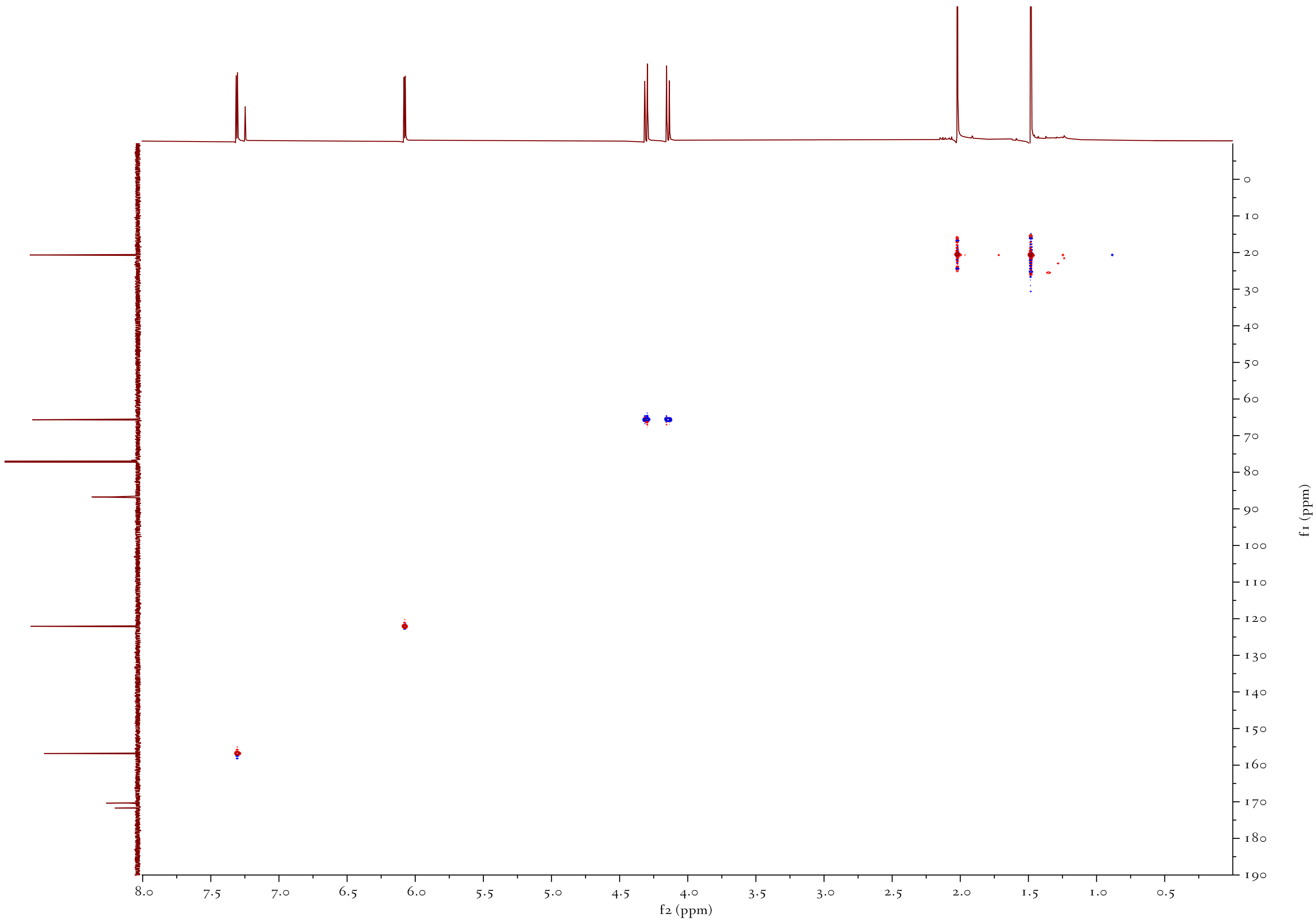

Figure S5. HSQC spectrum of **1**. (600 and 150 MHz, $CDCl_3$).

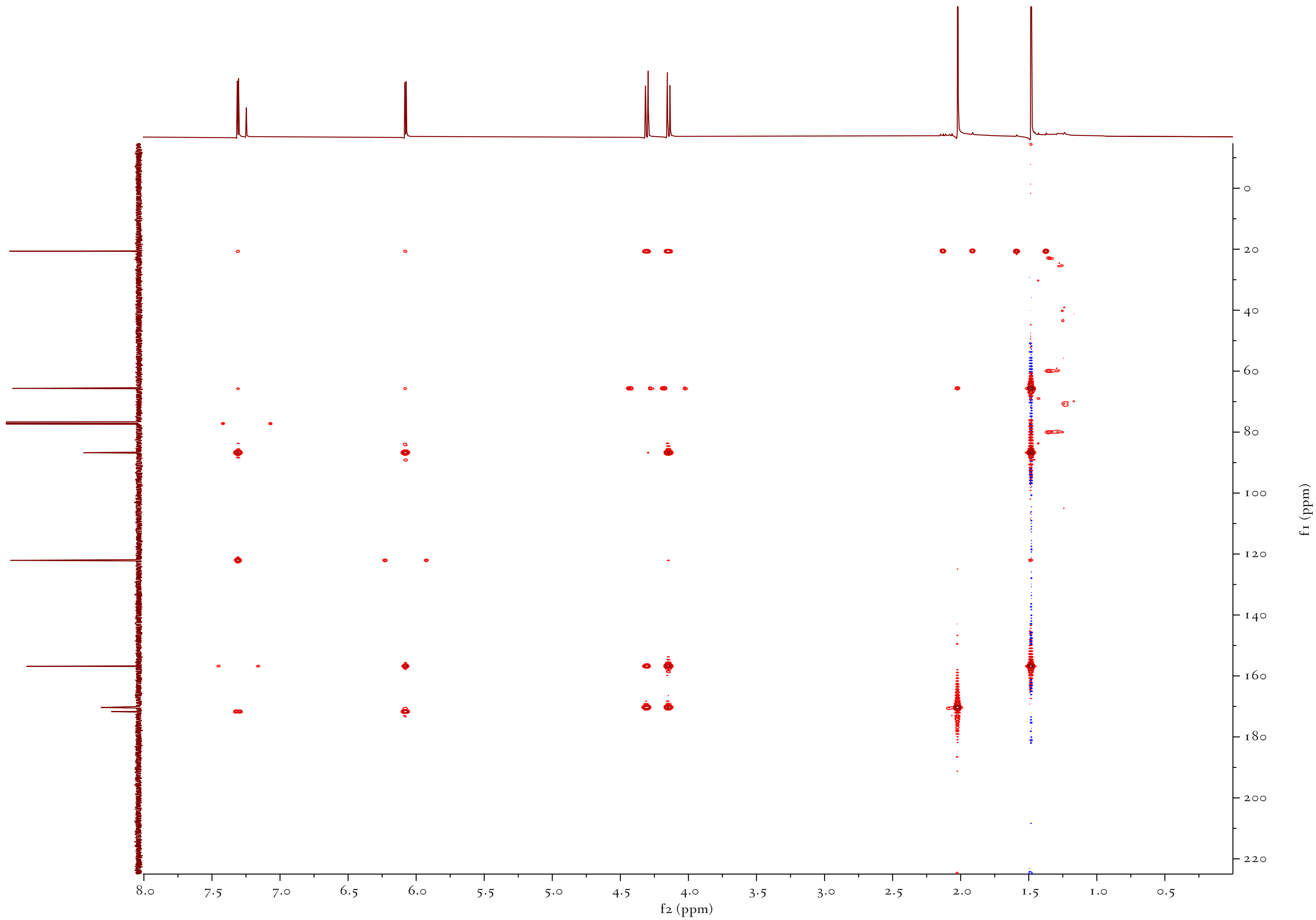

Figure S6. HMBC spectrum of **1**. (600 and 150 MHz, $CDCl_3$).

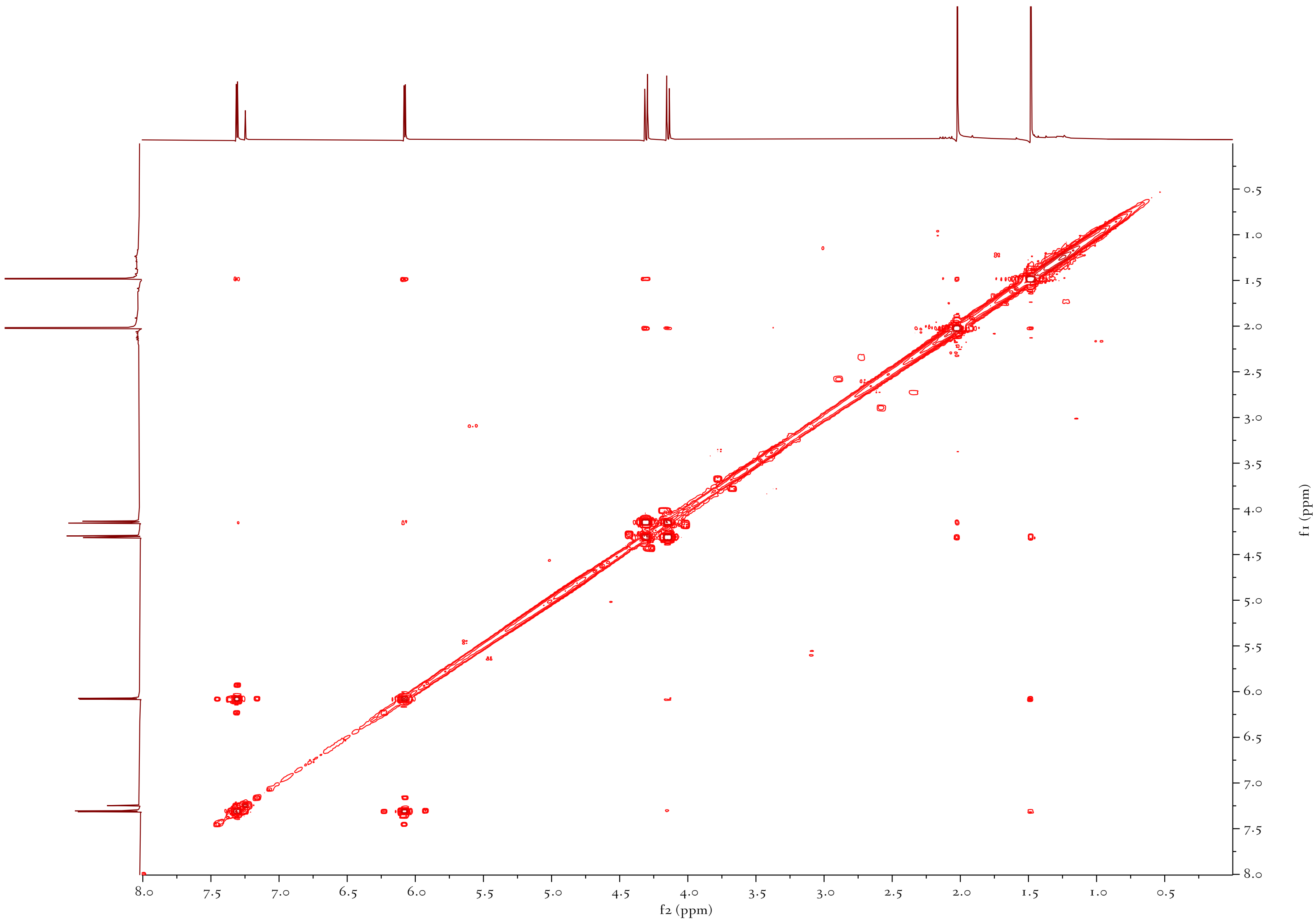

Figure S7. COSY spectrum of **1**. (600 MHz, $CDCl_3$).

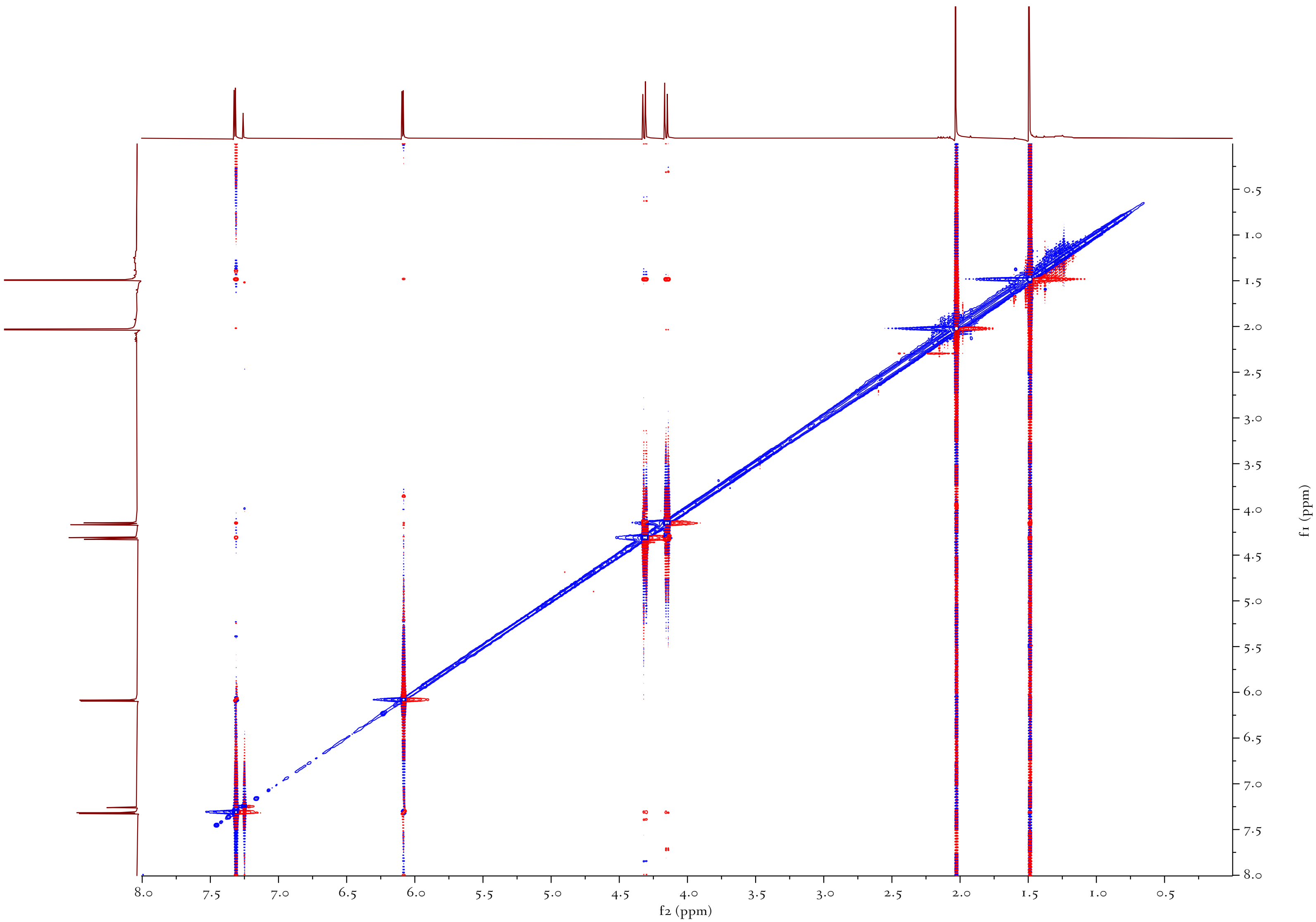

Figure S8. NOESY spectrum of **1**. (600 MHz, $CDCl_3$).

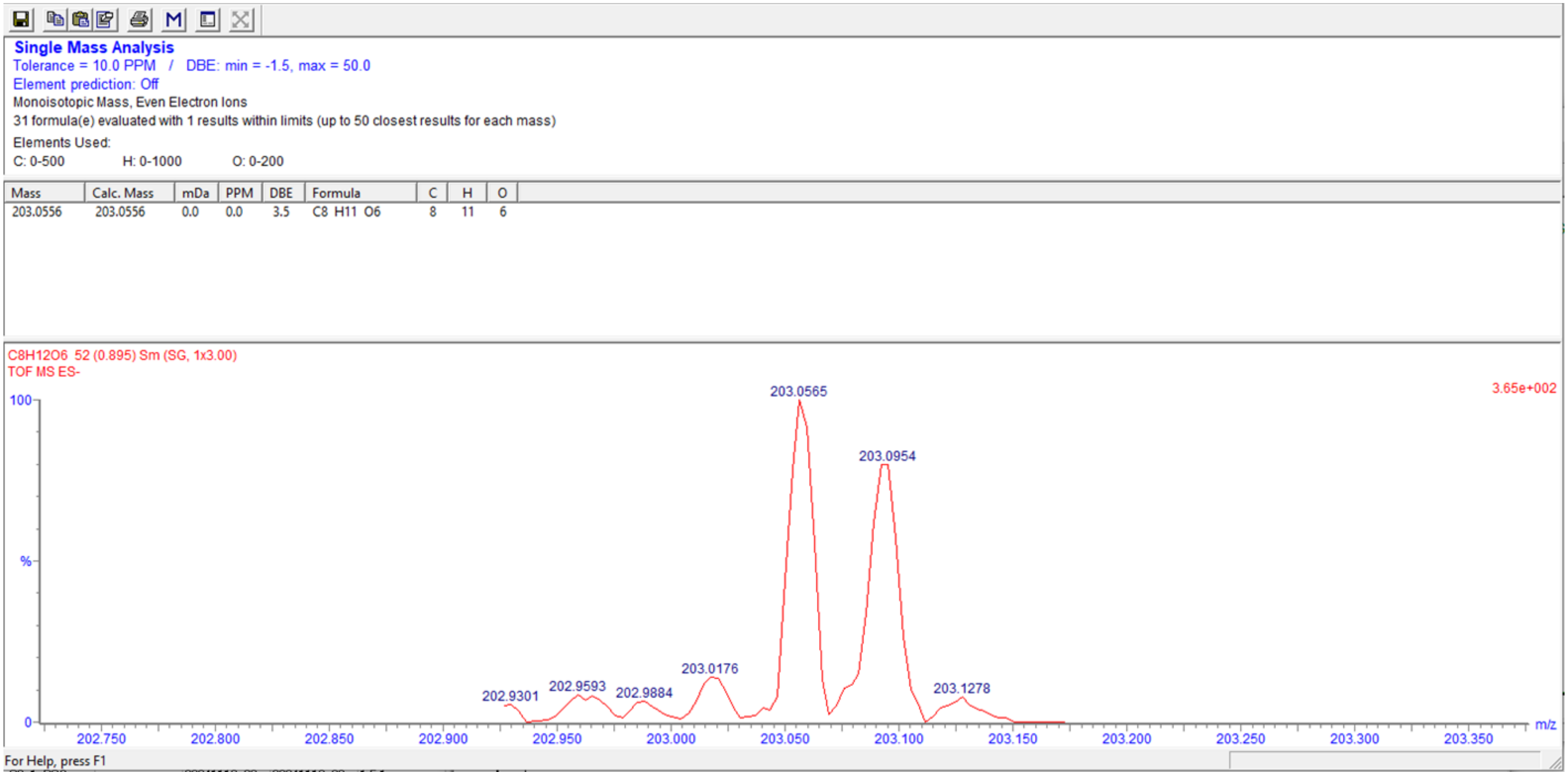

Figure S9. HRESIMS spectrum of **1**.

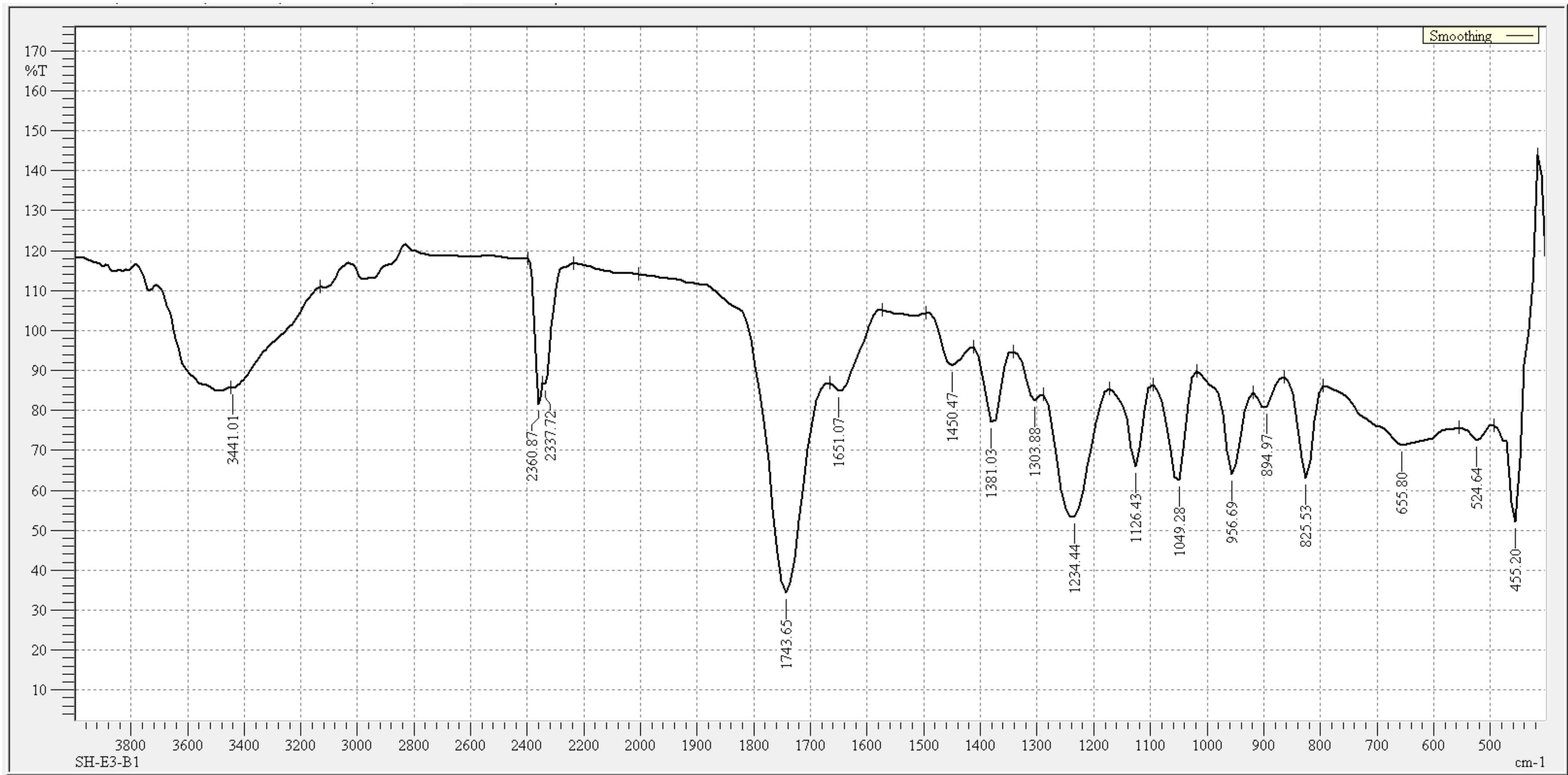

Figure S10. Infrared (IR) spectrum **1**.

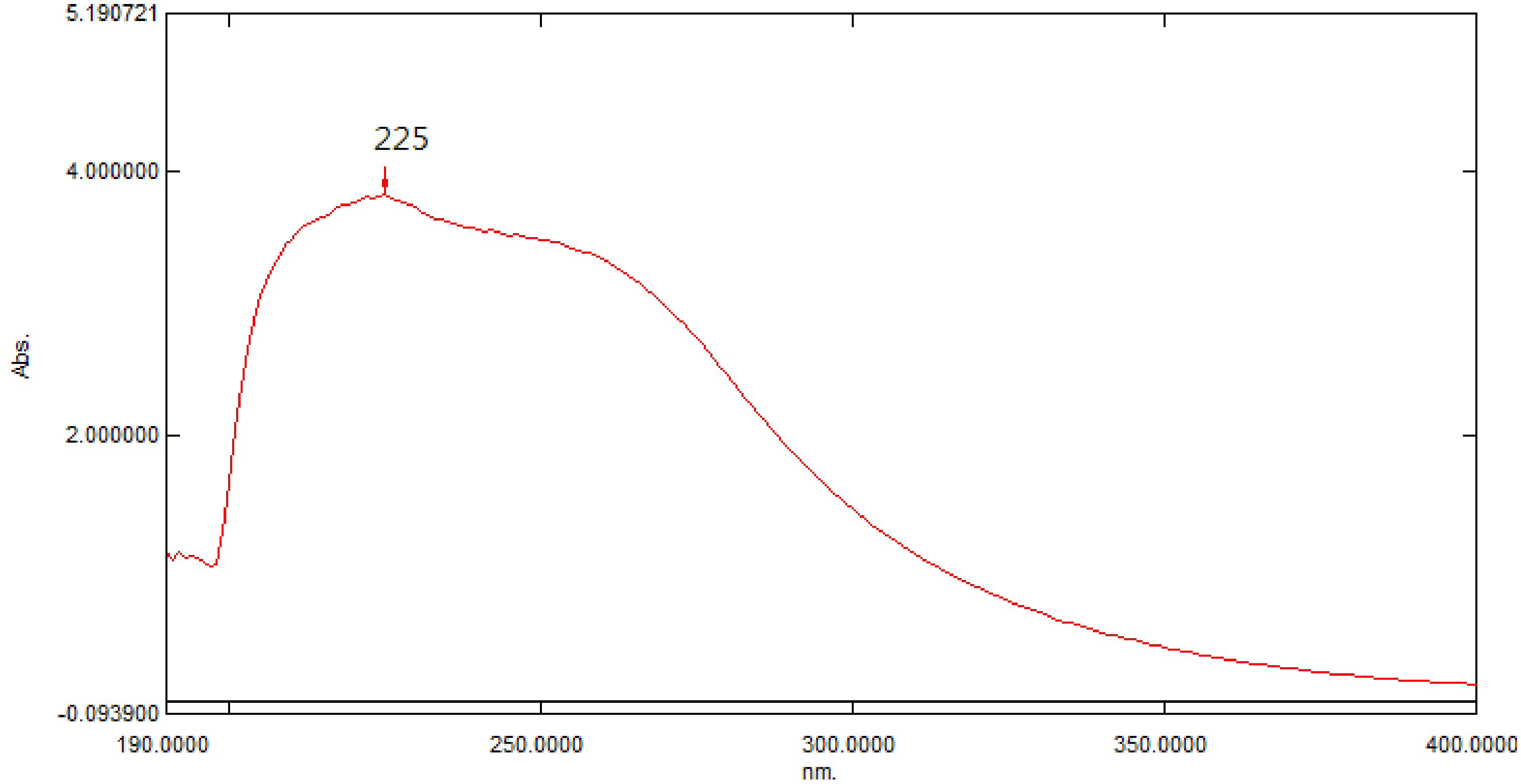

Figure S11. Ultraviolet (UV) spectrum **1**.

1.2. NMR, HRESIMS, IR, and UV spectra of 2.

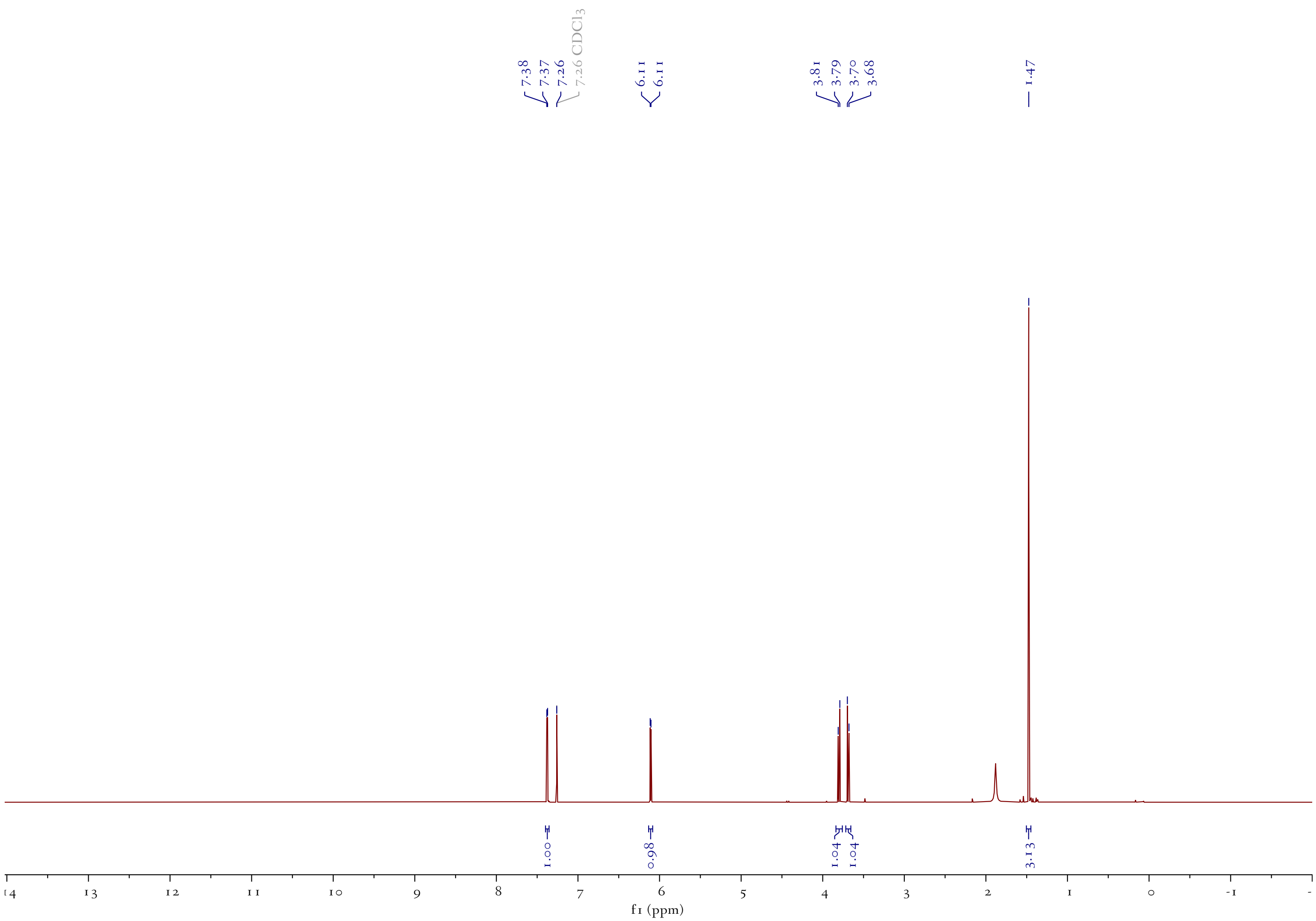

Figure S12. ^{1}H NMR spectrum of **2**. (600 MHz, $CDCl_3$).

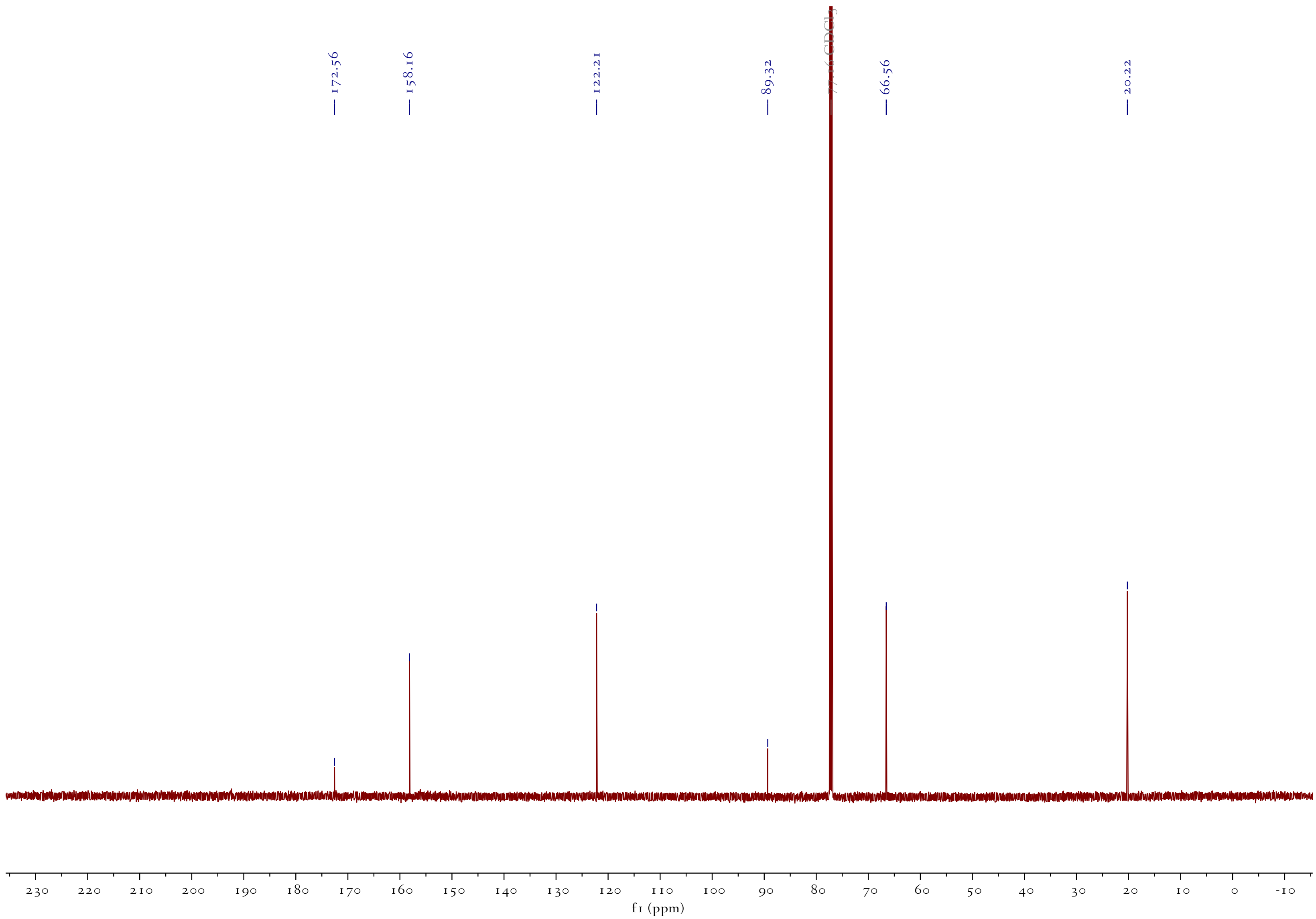

Figure S13. ^{12}C NMR spectrum of **2**. (150 MHz, $CDCl_3$).

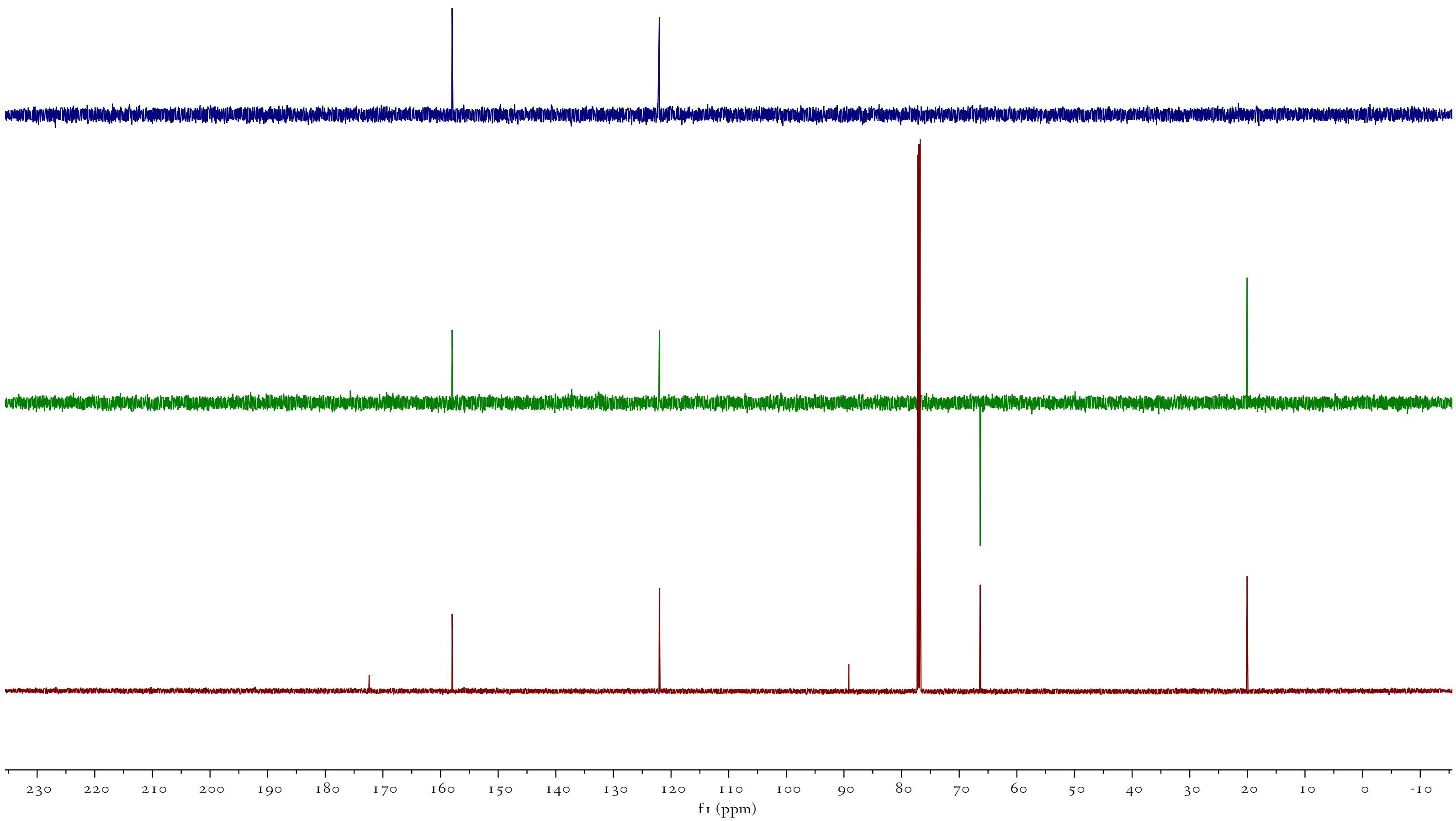

Figure S14. DEPT spectra of **2**. (150 MHz, $CDCl_3$).

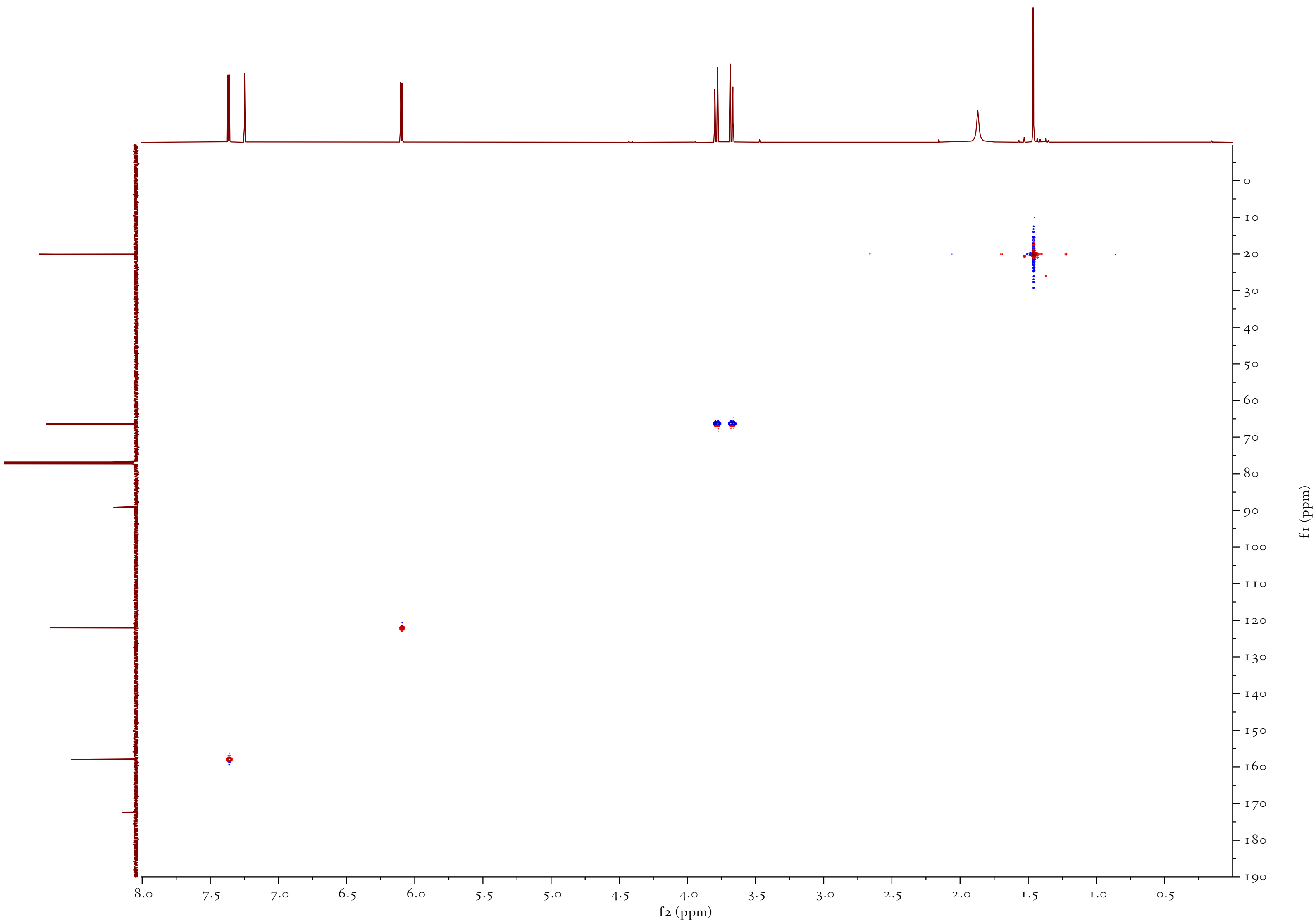

Figure S15. HSQC spectrum of ... (**2**) (600 and 150 MHz, $CDCl_3$).

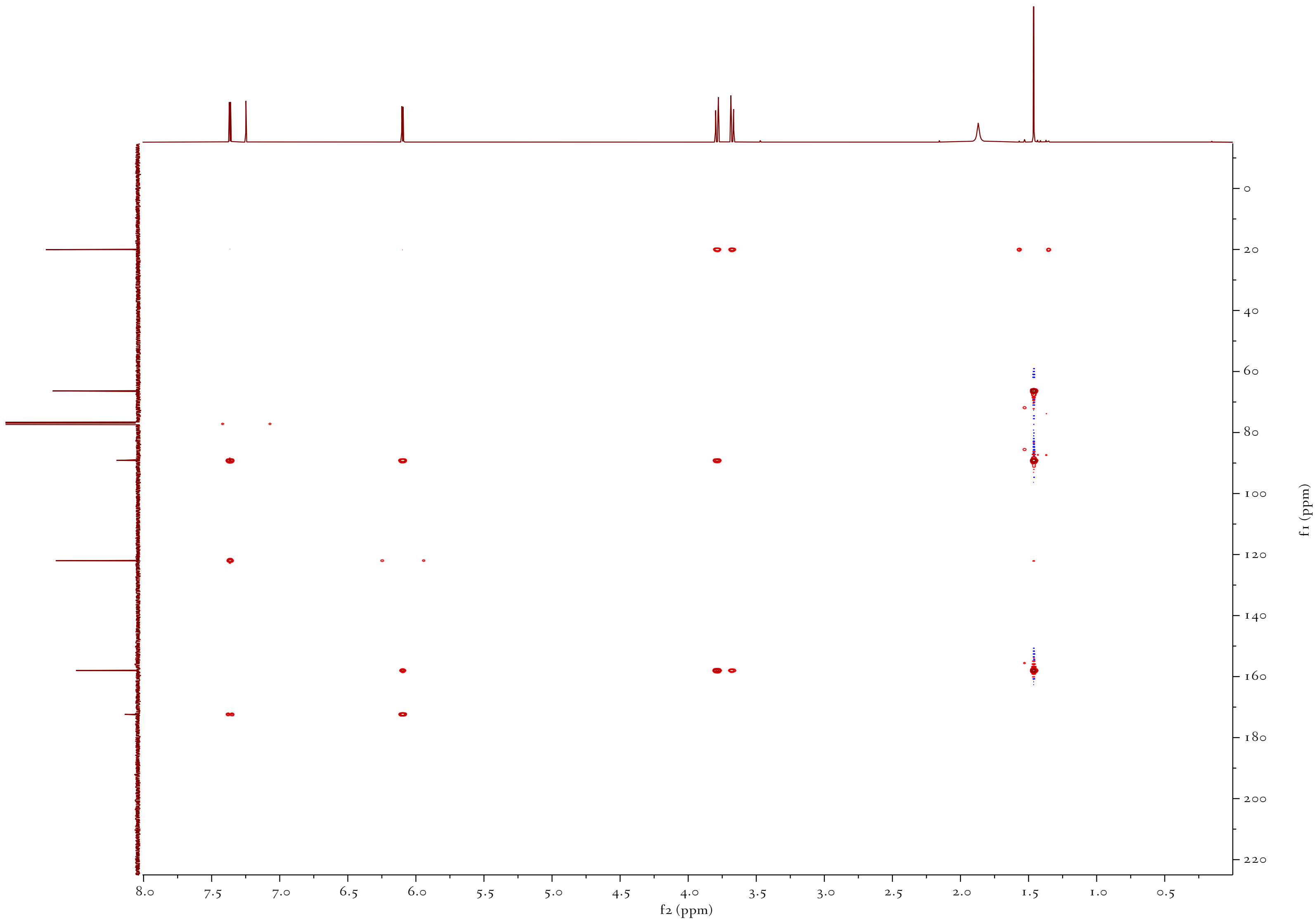

Figure S16. HMBC spectrum of **2**. (600 and 150 MHz, $CDCl_3$).

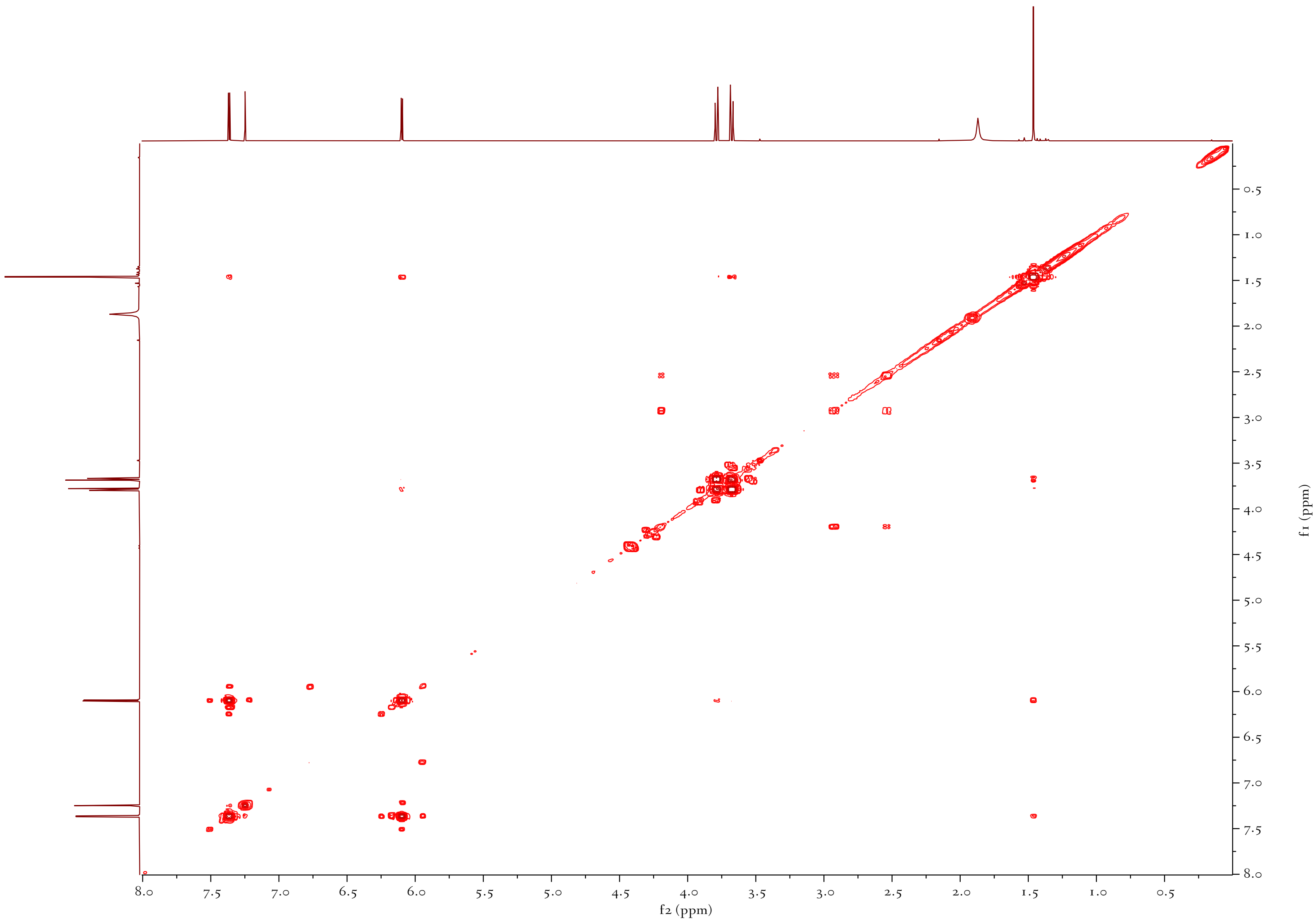

Figure S17. COSY spectrum of **2**. (600 MHz, $CDCl_3$).

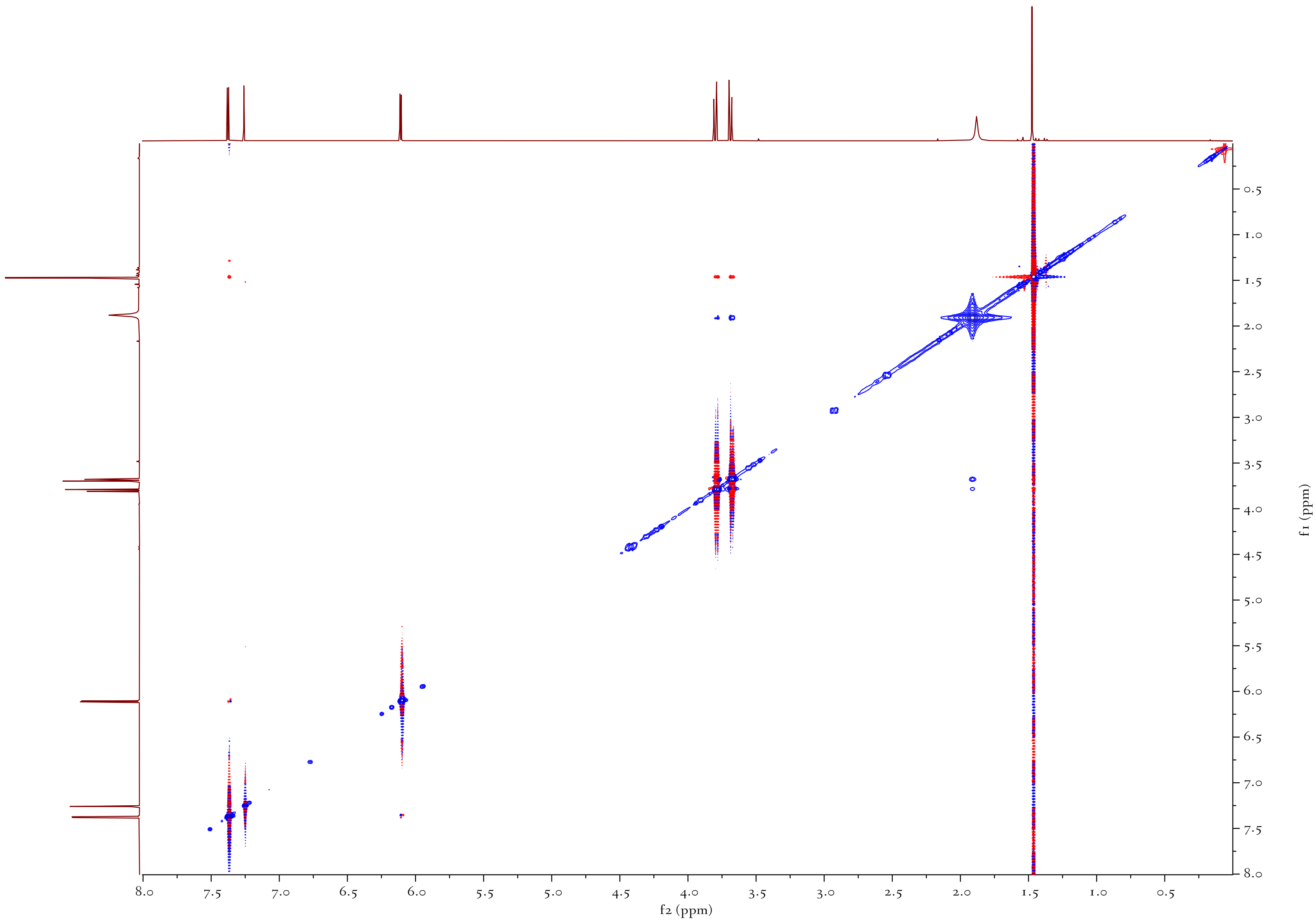

Figure S18. NOESY spectrum of **2**.(600 MHz, $CDCl_3$).

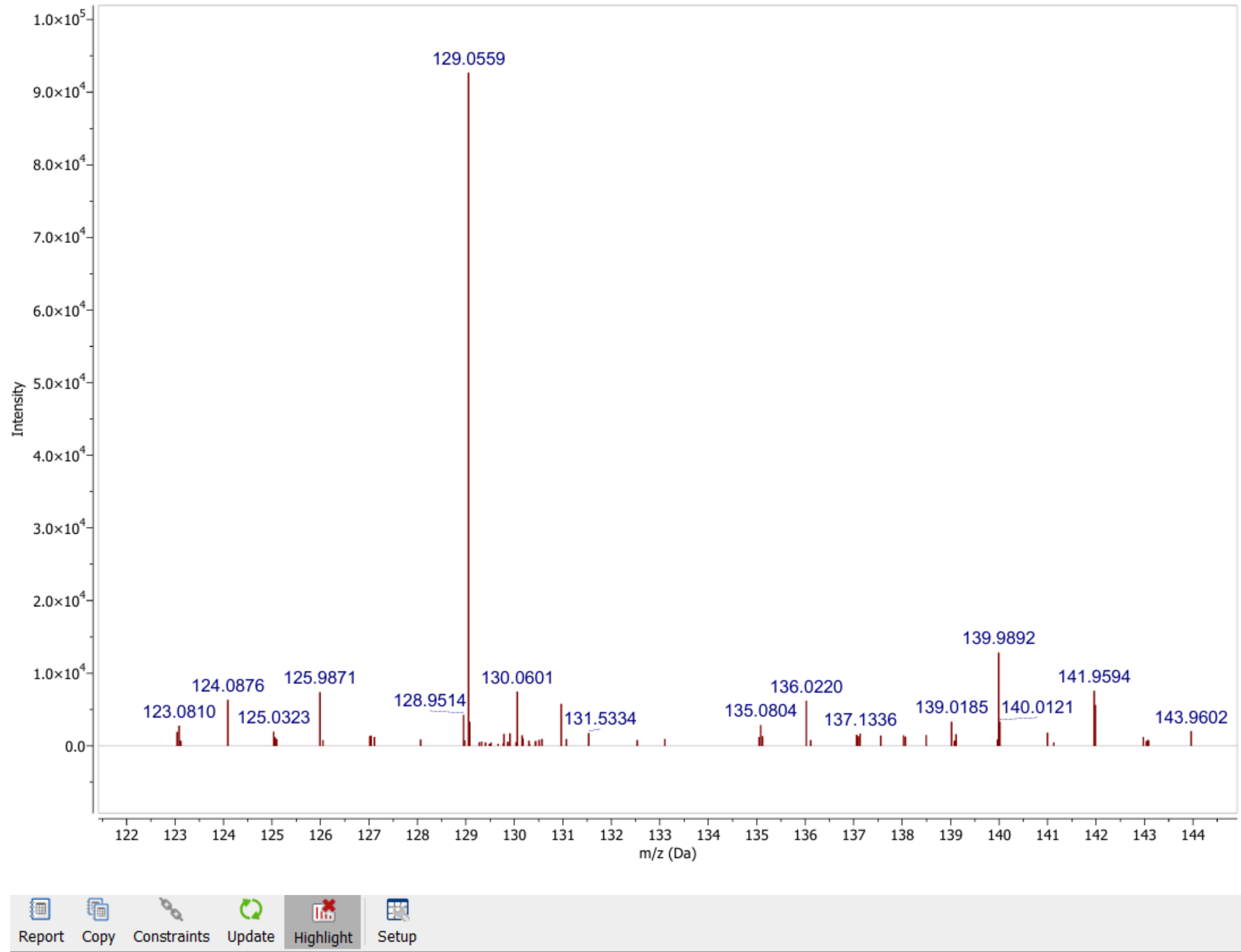

	Formula	Calculated Mass	Target Mass	Double Bond Equivalence	Absolute Error (ppm)	Error (mDa)	Error (ppm)	Fitness
1	$C_6H_9O_3$	129.05462	129.05594	2.5	10.20	1.32	10.20	1.000

Figure S19. HRESIMS spectrum of **2**.

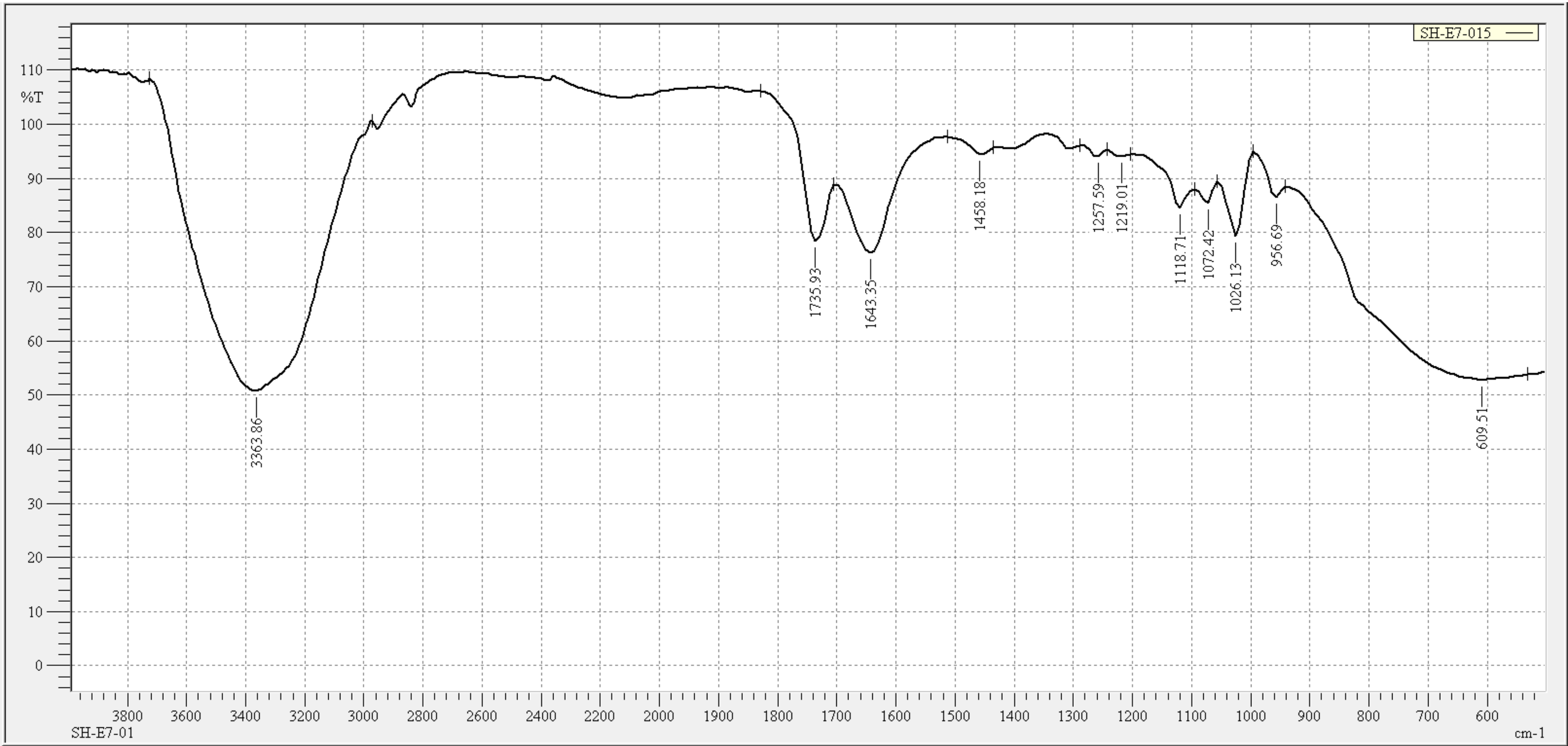

Figure S20. Infrared (IR) spectrum **2**.

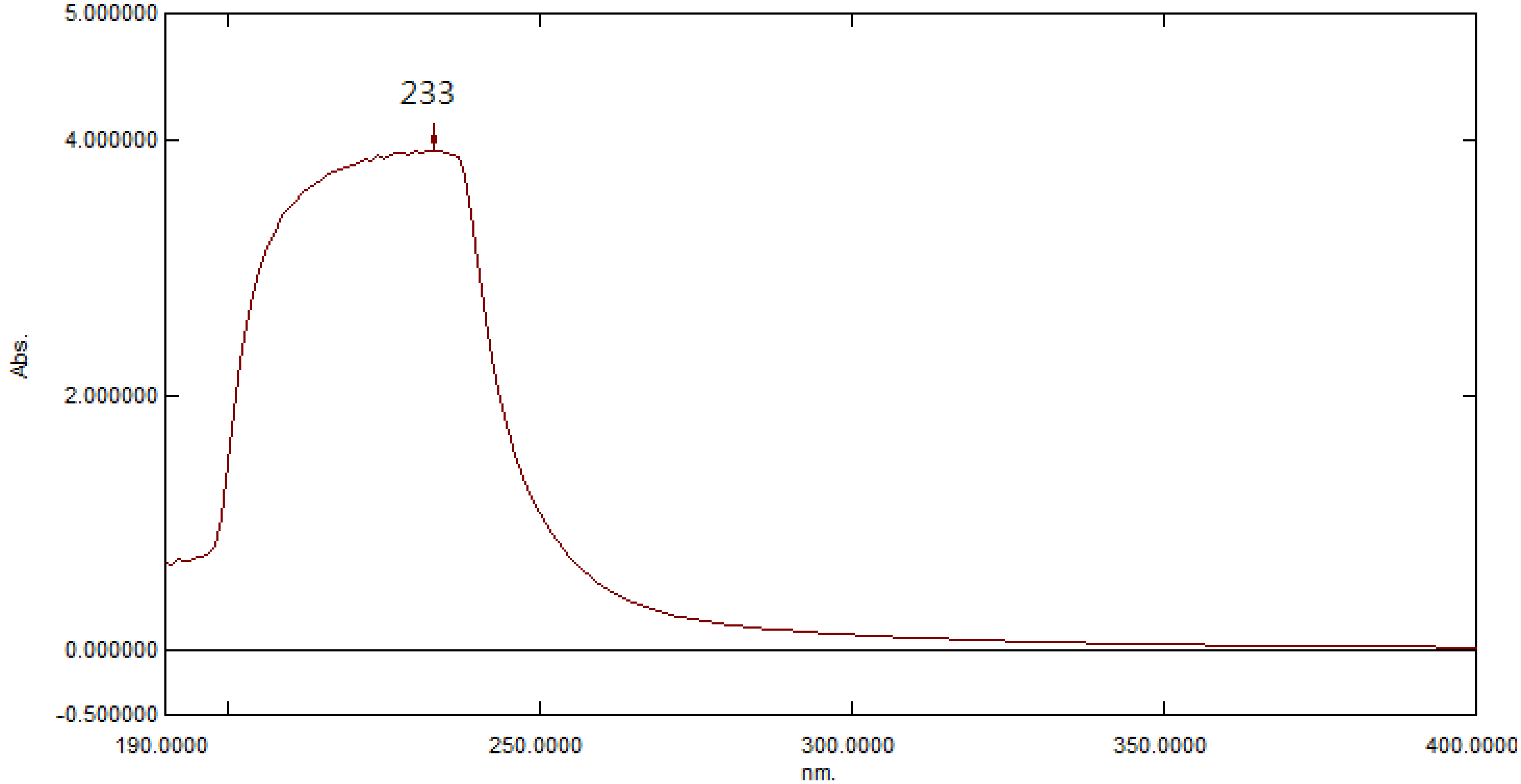

Figure S21. Ultraviolet (UV) spectrum **2**.
